# Population structure and pathogen interaction of *Escherichia coli* in freshwater: Implications of land‐use for water quality and public health in Aotearoa New Zealand

**DOI:** 10.1111/1758-2229.13319

**Published:** 2024-08-02

**Authors:** Adrian L. Cookson, Meg Devane, Jonathan C. Marshall, Marie Moinet, Amanda Gardner, Rose M. Collis, Lynn Rogers, Patrick J. Biggs, Anthony B. Pita, Angela J. Cornelius, Iain Haysom, David T. S. Hayman, Brent J. Gilpin, Margaret Leonard

**Affiliations:** ^1^ AgResearch Limited Hopkirk Research Institute Palmerston North New Zealand; ^2^ mEpiLab, School of Veterinary Sciences Massey University Palmerston North New Zealand; ^3^ Institute of Environmental Science and Research Kenepuru Science Centre Porirua New Zealand; ^4^ Institute of Environmental Science and Research Christchurch New Zealand; ^5^ School of Mathematical and Computational Sciences Massey University Palmerston North New Zealand; ^6^ School of Natural Sciences Massey University Palmerston North New Zealand

## Abstract

Freshwater samples (*n* = 199) were obtained from 41 sites with contrasting land‐uses (avian, low impact, dairy, urban, sheep and beef, and mixed sheep, beef and dairy) and the *E. coli* phylotype of 3980 isolates (20 per water sample enrichment) was determined. Eight phylotypes were identified with B1 (48.04%), B2 (14.87%) and A (14.79%) the most abundant. *Escherichia marmotae* (*n* = 22), and *Escherichia ruysiae* (*n* = 1), were rare (0.68%) suggesting that these environmental strains are unlikely to confound water quality assessments. Phylotypes A and B1 were overrepresented in dairy and urban sites (*p* < 0.0001), whilst B2 were overrepresented in low impact sites (*p* < 0.0001). Pathogens ((*Salmonella*, *Campylobacter*, *Cryptosporidium* or *Giardia*) and the presence of diarrhoeagenic *E. coli*‐associated genes (*stx* and *eae*) were detected in 89.9% (179/199) samples, including 80.5% (33/41) of samples with putative non‐recent faecal inputs. Quantitative PCR to detect microbial source tracking targets from human, ruminant and avian contamination were concordant with land‐use type and *E. coli* phylotype abundance. This study demonstrated that a potential recreational health risk remains where pathogens occurred in water samples with low *E. coli* concentration, potential non‐recent faecal sources, low impact sites and where human, ruminant and avian faecal sources were absent.

## Graphical abstract

Eight *E. coli* phylotypes were identified in freshwater samples; A and B1 were prevalent in dairy and urban areas, while B2 was common in low‐impact sites. Pathogens were found in 89.9% of samples, posing a potential recreational health risk even with low *E. coli* concentrations and absence of common faecal sources.

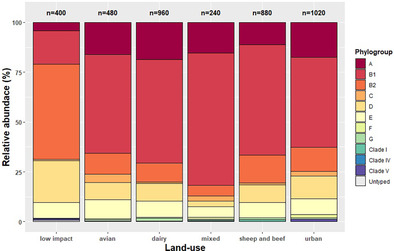

## INTRODUCTION

*Escherichia coli* is used by many water managers as a reliable indicator of faecal contamination in water quality assessments (Till et al., 2008). This bacterium, which normally inhabits the intestines of warm‐blooded animals (Tenaillon et al., 2010), serves as a sentinel faecal indicator bacterium (FIB), providing valuable insights into the potential presence of harmful pathogens in water sources (Cookson et al., 2022; Hörman et al., 2004; Nowicki et al., 2021; Weller et al., 2020). The use of *E. coli* as an indicator is rooted in its prevalence and high concentration in the gastrointestinal tracts of humans and other animals, making it a practical and efficient marker for assessing water safety (Touchon et al., 2020).

Water quality is a critical concern for public health, as contaminated water can harbour a variety of pathogens such as Shiga toxin‐producing *Escherichia coli* (STEC), *Campylobacter*, *Salmonella* and *Cryptosporidium* and *Giardia* that pose serious risks to human well‐being (Hörman et al., 2004; Weller et al., 2020). Faecal contamination is a key focus in water quality assessments due to its association with waterborne diseases; while there are numerous bacteria, viruses, and protozoa that may indicate faecal contamination, *E. coli* is favoured as it is relatively easy and cost‐effective to culture and detect. Standardized methods such as membrane filtration and Colilert (IDEXX) allow for efficient and reliable identification and quantification of *E. coli* to assess public health risk (Till et al., 2008). Moreover, *E. coli* has a short incubation period, meaning that its presence in water samples can be detected relatively quickly (12–24 h). This characteristic is crucial for timely responses to potential waterborne outbreaks, allowing water managers to take prompt action to protect public health. The presence of *E. coli* in water is indicative of recent faecal contamination, suggesting a higher likelihood of the presence of other, potentially harmful, waterborne pathogens which often are slower growing and require more complex culture detection methods (Devane et al., 2020). An association between *E. coli* and the risk of gastroenteric illness (Wiedenmann et al., 2006) makes it a valuable tool for assessing the overall safety of water sources (Till et al., 2008).

Recent studies utilizing high resolution genomics have demonstrated that several benign ‘cryptic’ *Escherichia* clades, indistinguishable from generic *E. coli* phenotypically, may also survive and persist in the environment (Cookson et al., 2022; Luo et al., 2011; Mire et al., 2022; Walk et al., 2009). Formerly identified as *E. coli* strains, *Escherichia whittamii* (Clade II) (Gilroy et al., 2021), *Escherichia ruysiae* (Clades III and IV) (van der Putten et al., 2021) and *Escherichia marmotae* (Clade V) (Liu et al., 2015) have been described as novel species, however, current culture‐based methods, used to enumerate *E. coli* as a proxy for faecal contamination and pathogenic microorganisms, cannot distinguish between environmental *Escherichia* clades and faecal strains (Walk, 2015). This lack of discrimination could lead to a potential overestimation of health risk using standard culture‐based methods of *E. coli* determination.

In addition to the naturalized *Escherichia* spp., whole genome sequencing of faecal *E. coli* has provided important phylogenetic insights into intra‐species variation with the identification of at least eight distinct phylotypes (Clermont et al., 2011; Clermont et al., 2013; Clermont et al., 2019). Paradoxically, some *E. coli*, particularly phylotypes B1 (Berthe et al., 2013; Nowicki et al., 2021; Power et al., 2005; Ratajczak et al., 2010; Touchon et al., 2020; Walk et al., 2007) and B2 (Cookson et al., 2022; Petit et al., 2017), have also been associated with prolonged survival and growth in environmental samples (e.g., water and sediment), where no obvious faecal contamination event has been noted. These observations have been described as relating to non‐recent or aged contamination events and typically refer to incidents involving faecal contamination into a particular water source occurring some time previously and not being of recent origin. Thus, there appear to be two groups of naturalized *Escherichia*; from environmental, and those from enteric sources, but the extent to which either or both confound microbial water quality assessments is unknown (Devane et al., 2020).

Measuring FIB offers insights into contamination levels but standard culture‐based methods are unable to identify faecal sources (Harwood et al., 2014; Tran et al., 2015). The implementation of molecular methods to identify host‐associated microbial source tracking (MST) markers can provide valuable insights into the faecal sources of pathogens impacting human health in water samples (Devane et al., 2018; Devane et al., 2019; Devane et al., 2020; Korajkic et al., 2019). Links with host‐associated faecal markers from humans, ruminants and avian sources can assist water managers with targeted mitigation strategies to reduce pathogen levels and for more accurate public health risk assessments.

In this study, we describe detailed analysis of *E. coli* phylotypes, freshwater pathogens, land‐use, and host‐associated (human, ruminant and avian) MST markers obtained from freshwater samples collected as part of the New Zealand Ministry for the Environment's (MfE) Freshwater Pathogen Quantitative Microbial Risk Assessment (QMRA) studies as an update to the 1998–2000 Freshwater Pathogen QMRA study (Till et al., 2008). In parallel with bacterial and protozoal pathogen detection data, the objectives of this study were to (i) examine the prevalence of the recently described *E. marmotae*, *E. ruysiae* and *E. whittamii* species; (ii) describe any relationship of phylotypes with *E. coli* concentration and land‐use; and (iii) assess the relationship between naturalized *E. coli* phylotypes B1 and B2 with non‐recent or aged faecal sources and local land‐use.

## EXPERIMENTAL PROCEDURES

### Water sampling sites

**FIGURE 1:**
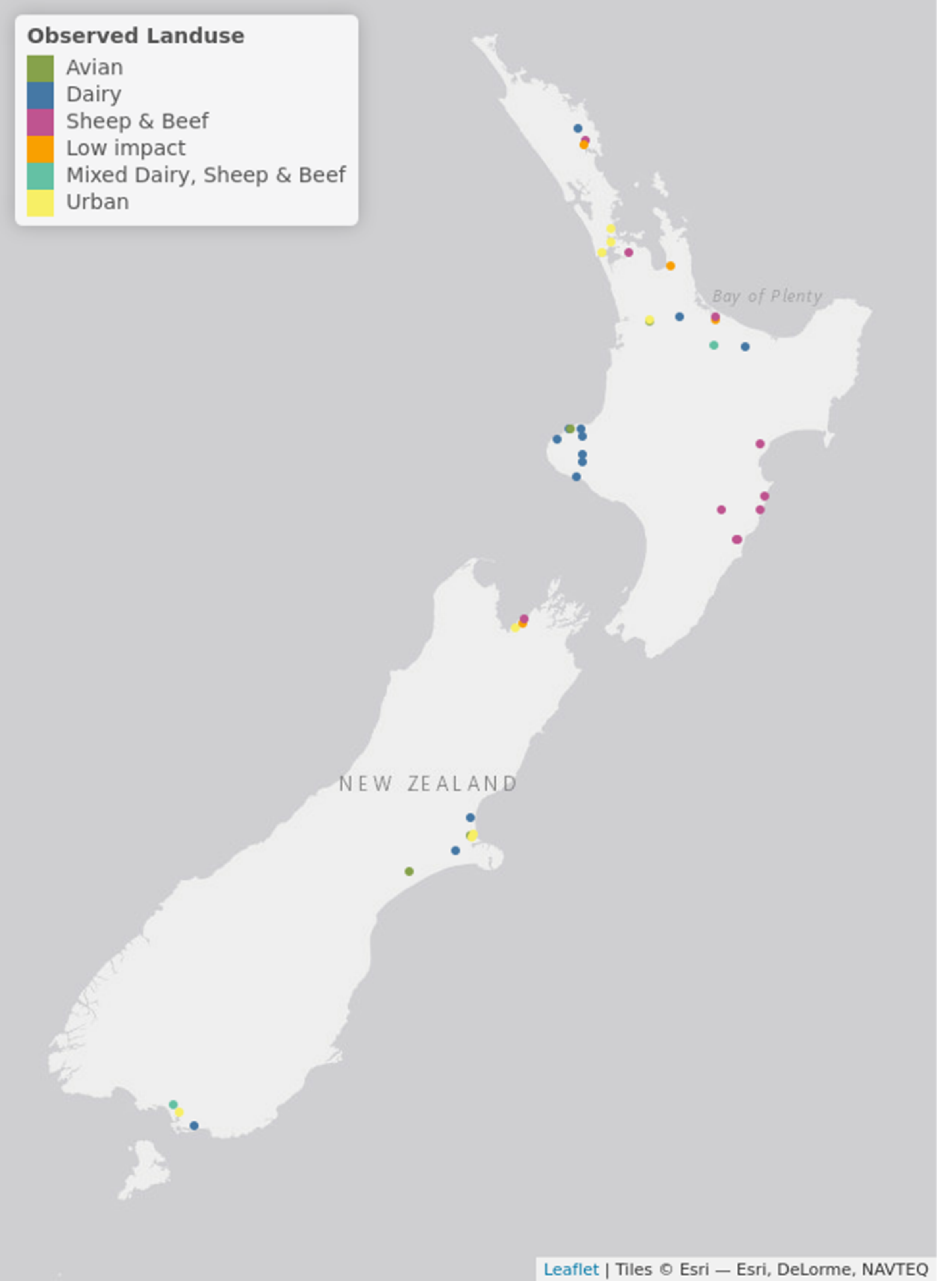
New Zealand water sampling sites and associated dominant land‐use included as part of this study.

The water samples for this study originated from 2022 to 2023 large‐scale MfE Freshwater Pathogen study collected as part of a wider study to update the 1998–2000 QMRA (Till et al., 2008). Freshwater samples were taken as part of routine monitoring programs by Council (local New Zealand environmental agency) staff in late spring to early winter (October 2022–July 2023) at 41 sites around New Zealand (Figure 1). Sampling was scheduled to provide national coverage of different land‐uses taking into account the logistics associated with long term sampling across nine different Councils at 41 sites.

### Catchment delineation and land‐use identification for freshwater sampling sites

A hydrological sub‐catchment for each sampling site was generated using ArcGIS Pro (v.2.6.4). In each respective catchment, the sampling location was used as a pour point in the catchment delineation analysis. The pour point specifies where water should ‘drain’ from the catchment, thereby giving the total land surface draining to the sampling point. The catchment sizes ranged from approximately 3–8 km^2^. Land cover data (Table S1) for each of the 41 sites was sourced from the Land Cover Database version 5.0, Mainland, New Zealand (Anon, 2020a) to assist with identifying the dominant land‐use for site categorization.

Sites with a history of elevated concentrations of *E. coli* were located across New Zealand and reflected different dominant land‐uses and impacts as designated by Councils: dairy (*n* = 13); sheep and beef (*n* = 10); mixed sheep and beef and dairy (*n* = 2); urban (*n* = 8); avian (*n* = 4). Four sites were designated as low impact with lower potential for faecal contamination (exotic/natural forest dominated control sites). Sample sites were designated as ‘avian’ using prior observational information provided by Councils which noted large numbers of avian faecal contamination events caused by wildfowl flocks or bird roosts. Generally, sampling occurred between 6 AM and 1.30 PM New Zealand standard time. Samples were collected 200 mm below the water surface using aseptic methods. Most samples sites were selected from non‐tidal areas, however, if samples sites affected by tidal influences were identified, they were sampled around low tide. If the water was too deep, samples were taken from the bank using an extendable pole. After collection, samples were cooled and transported overnight with frozen packs to keep water samples chilled for FIB, MST and pathogen analyses to the Institute of Environmental Science and Research (ESR, Christchurch, New Zealand). Samples for protozoan analyses were sent overnight to MicroAquaTech, Massey University (New Zealand).

### Campylobacter isolation and detection

For quantitative analysis of *Campylobacter* by Most Probable Number (MPN) enrichment, water volumes of 1 × 1000 ml, 3 × 10 ml, 3 × 1 ml and 3 × 0.1 ml were analysed (APHA, 2017a; ISO, 2017; Medeiros & Hoffman, 2002) and cultured using Preston broth (Fort Richard Laboratories Ltd., Auckland, New Zealand) as described in the Data S1. Preston broth culture was plated onto modified charcoal‐cefoperazone‐deoxycholate agar (mCCDA) plates (Fort Richard) and incubated at 41.5 ± 1°C for 44 (±4) h, and then 2–4 putative *Campylobacter* colonies were transferred to Columbia Blood Agar (CBA) plates (Merck, Darmstadt, Germany) and incubated as before. Where putative *Campylobacter* colonies were observed on CBA, conventional PCR (Wong et al., 2004) was performed on DNA extracted from the original Preston broth culture to identify *C. coli*, *C. jejuni* and thermophilic *Campylobacter*.

### Salmonella isolation and detection

For presence/absence analysis of *Salmonella*, 1 L of water was filtered through 0.45 μm (47 mm diameter) mixed cellulose ester membrane filters (Millipore, Merck) and placed into 25 ml of buffered peptone water (BPW) broth (Fort Richard Laboratories Ltd) and incubated at 37 ± 1°C for 18 ± 2 h as described in the Data S1. Isolates that were consistent with *Salmonella* were tested for the presence of *Salmonella* O‐ and H‐antigens by slide agglutination using polyvalent antisera (Remel, ThermoFisher Scientific, Vilnius, Lithuania) and metabolic characteristics (Microbact GNB 12A and 12B kits in combination, ThermoFisher Scientific).

### Cryptosporidium and giardia isolation and detection

Protozoa were detected from 30 L water samples according to USEPA Methods (USEPA, 2005) as described in the Data S1. Immunomagnetic separation (IMS) using a Dynabeads GC‐Combo kit (ThermoFisher Scientific) and immunofluorescent staining for *Cryptosporidium* oocysts and *Giardia* cysts was performed using the EasyStain kit (BioPoint Pty Ltd.).

### Enumeration and storage of faecal indicator bacteria

*Escherichia coli* were analysed in freshwater using Colilert Quanti‐Tray assays (100 ml of a 1 in 10 dilution of water sample, incubated at 35 ± 0.5°C for 24 to 28 h) (IDEXX Laboratories, Inc., Maine) (APHA, 2017b). This provides a detection range of <10 Most Probable Number (MPN)/100 ml up to >24,200 MPN/100 ml. Wells that were yellow in colour and fluoresced when exposed to UV light, indicative of the presence of *E. coli*, were identified with a hand‐held UV unit (EA‐160/FE, Spectroline, New York). Between 3 October 2022 and 3 July 2023, positive Colilert 2000 trays (IDEXX, New Zealand) from 298 water samples stored at 4°C after testing was complete were transported (maintained at chilled temperature during overnight transit) in single weekly batches to AgResearch (Hopkirk Research Institute, Palmerston North) from ESR, Christchurch. For each individual Colilert tray, wells that fluoresced when exposed to UV light, indicative of the presence of *E. coli* were identified with a hand‐held UV unit (EA‐160/FE, Spectroline, New York), carefully opened using a sterile low‐gauge needle, and 150 μl (small wells) or 200 μl (large wells) of growth removed from each after pipetting up and down three times to homogenize the bacterial suspension in each well. Bacterial culture from the fluorescent wells of each individual Colilert plate was pooled and centrifuged at 5000×*g* for 10 min at 4°C using a Multifuge X3R (Thermo Fisher Scientific). The supernatant was discarded, the pellet resuspended in 1 ml EC broth and transferred to a 1.5 ml tube and centrifuged at 16,200×*g* for 2 min. The supernatant was discarded, the bacterial pellets were resuspended in 1 ml EC broth (Fort Richard Laboratories Ltd.) and added to cryovials containing glycerol (final concentration 30% v/v) and stored at −80°C.

Colilert trays (*n* = 298) were sub‐grouped to balance *E. coli* MPN/100 ml concentrations of <100 (low), 101 to 1000 (medium), and >1000 (high) in a 1:2:1 ratio across the 41 sample sites, where possible, to focus on samples requiring regulatory authority action in New Zealand: (i) 260 *E. coli* MPN/100 ml to spur water managers to identify sources of faecal contamination for mitigation purposes and (ii) 540 *E. coli* MPN/100 ml to inform the public that the site is unsuitable for primary contact recreational use (Anon, 2020b). Subsequent isolation of *E. coli* was undertaken from a selection of 199 frozen glycerol stocks across all 41 sample sites on ECC CHROMagar plates (Fort Richard Laboratories Ltd.). Plates were incubated at 35°C for 18 to 21 h after which 20 well‐spaced, individual blue colonies (presumptive *E. coli*) from each water enrichment (stored in glycerol) were selected and sub‐cultured onto MacConkey Agar plates (Fort Richard Laboratories Ltd.) and incubated as previously described. A crude DNA sample for further DNA analysis was prepared for each of the 3980 isolates by resuspending a single well‐spaced colony in 400 μl of sterile milliQ water, heated at 100°C for 10 min in a heating block and stored at −20°C. Another single well‐spaced colony was resuspended in 1.5 ml of sterile Brain heat infusion (BHI) broth (Oxoid) containing glycerol (30% v/v) and stored at −80°C. In total, 3980 presumptive *E. coli* were stored from 199 water samples.

### Multiplex PCRs for Escherichia species and E. Coli phylotypes

Each of the boiled lysate preparations (*n* = 3980) were used in individual PCR reactions to determine the *E. coli* phylotype or *Escherichia* cryptic Clades (I to V) using an extended quadruplex PCR phylotype assignment method (Clermont et al., 2011, 2013). Allele specific multiplex PCR for the identification of phylotypes was carried out as described in the Data S1. Clade I to V PCRs were carried out according to Clermont et al (2011).

### *Real‐time PCR (RT‐PCR) for detection of diarrhoeagenic* E. col*i genes*

Real‐time multiplex PCR (RT‐PCR) assays were performed on an Applied Biosystems ViiA7 Real‐time PCR System (Life Technologies) targeting the virulence genes *stx*1 and *stx*2 from Shiga toxin‐producing *E. coli* (STEC), and intimin adherence (*eae*) gene from enteropathogenic *E. coli* (EPEC) and some STEC serogroups, as markers for the presence of pathogenic *E. coli* (Clarke, 2001; Nataro & Kaper, 1998), and the *E. coli* 16S rRNA gene as an internal control. Positive samples were described as those having quantification cycle thresholds (Cq) of <35.

### Additional metadata

Rainfall data for water samples in the preceding 24, 48 and 72 h, was recorded by Council staff from each site and visit and was included in analysis. Average flow data (m^3^/s) from the preceding 24 h prior to sampling were provided by Council staff.

### Microbial source tracking markers

Freshwater samples were filtered (in duplicate) through a 0.45 μm mixed cellulose ester membrane filters (Millipore, France) and DNA extracted as described in the Data S1. Quantitative PCR (qPCR) analysis was undertaken on a LightCycler 480 (Roche Diagnostics Ltd, CA). The PCR targets are presented in Table 1 with PCR protocols and conditions outlined by Devane et al. (2013) and described in the Data S1. Each qPCR run included negative and positive controls, and standard curves of the appropriate target. The amplification efficiency of the PCR assays was considered acceptable at >90% for qPCR targets, and the coefficient of determination (*r* ^2^) at ≥0.92 for assays.

**TABLE 1:** Target bacterial genes and methods for qPCR.

Faecal source host/target	Microbial target	Type of qPCR assay (references)
General	Bacteroidales 16S rRNA (GenBac3)	Probe‐based (Siefring et al., 2008)
Human	Duplex qPCR of *Bacteroides* HF183 and crAssphage CPQ_056 *Bifidobacterium adolescentis* (BiADO)	Probe‐based (Ahmed et al., 2019) SYBR Green (Matsuki et al., 2004)
Ruminant	Bacteroidales 16S rRNA (BacR)	Probe‐based (Reischer et al., 2006)
Avian	GFD—Unclassified *Helicobacter* 16S rRNA gene	SYBR Green (Green et al., 2012)

### Statistical analysis

Statistical analysis was performed using R version 3.6.2 (R Development Core Team, 2018), and ggpubr (Kassambara, 2020); figures were produced using the package ggplot2 (Wickham, 2009). Shannon diversity (total number of *E. coli* phylotypes and *Escherichia* species) per water sample was undertaken using the R package ‘vegan’ version 2.5‐6 (Oksanen et al., 2020).

To test the effects of pathogens or pathogen‐associated genes (*Salmonella*, *Campylobacter*, *eae* gene, *stx* gene, *Cryptosporidium*, *Giardia*, Any Bacteria, Any Protozoa, Any Pathogen), lme4 (Bates et al., 2015) was used to fit binomial generalized linear mixed effect models on the pathogen presence or absence data, to test associations with, for example, *E. coli* concentration MPN/100 ml (log_10_MPN) or *E. coli* phylotype (total 0–20) from each water sample determined using PCR. Individual “Site” was included in the model as a random effect. ‘Any Pathogen’ refers to the presence/absence of the *Salmonella*, *Campylobacter*, *Cryptosporidium* and *Giardia* using culture or microscopy methods, and the detection of *stx* (Shiga toxin) and *eae* (intimin) genes commonly found in diarrhoeagenic *E. coli*, using RT‐PCR. Previous study has shown that unlike other *E. coli* subtypes, phylotype B1 may persist and be stably maintained in freshwater (Berthe et al., 2013; Ratajczak et al., 2010; Touchon et al., 2020; Walk et al., 2007) but its presence may still have implications for the quality and safety of the affected area. Thus, the relative abundance of this ‘generalist’ *E. coli* was used for exploratory purposes to test the novel assumption where B1 was used as a proxy for non‐recent faecal contamination when identified in the presence of reduced numbers of the other *E. coli* phylotypes expected in fresh contamination. Based on this hypothesis, each individual water sample was grouped by abundance of phylotype B1 identified from the 20 isolates to represent low (<10 B1 isolates), intermediate (10–14 B1 isolates), and high (≥15 B1 isolates) for inclusion in models. Linear mixed effects models were also used to examine the relationship between for example, *E. coli* phylotypes/*Escherichia* cryptic clades to examine the association with faecal source data. To assess whether there was a difference in phylotype profiles across samples by land‐use, a distance‐based analysis of variance (PERMANOVA) was performed including site as a random effect nested within land‐use (Anderson, 2001). *p* values of <0.05 were deemed as significant.

## RESULTS

An extended longitudinal study was undertaken over 39 weeks from October 2022 to July 2023. Visits were made to 41 sites (4 avian, 13 dairy, 4 low impact, 2 mixed—sheep, beef and dairy, 10 sheep and beef and 8 urban). On average, sites were visited on five separate occasions (min 1, max 9); two sites were visited only once as replacements for inaccessible sites due to the impacts of Cyclone Gabrielle (13 February 2023), which temporarily restricted access to the original selected sites. Following selection across low/medium/high *E. coli* concentrations (at 1:2:1 ratio) and across sample sites, 199 Colilert trays were taken forward for further analysis of 20 *E. coli* isolates from each tray where 45 (22.6%) were low, 106 (53.3%) medium and 48 (24.1%) high *E. coli* MPN/100 ml. *E. coli* were detected from all 199 water samples analysed in detail at a geometric mean of 379 MPN/100 ml (median 330 MPN/100 ml) and a geometric mean of 349 MPN/100 ml for all 298 samples received (median 315 MPN/100 ml); urban sites had the highest average *E. coli* contamination rates (geometric mean 813MPN/100 ml). Compared to low impact sites (geometric mean 91 MPN/100 ml), there was no significant difference in average *E. coli* concentrations for avian (geometric mean 166 MPN/100 ml, *p* = 0.43) and mixed impact sites (geometric mean 259 MPN/100 ml, *p* = 0.27), but average *E. coli* contamination rates for dairy (geometric mean 597 MPN/100 ml, *p* = 0.004), sheep and beef (geometric mean 318 MPN/100 ml, *p* = 0.046) and urban (*p* = 0.001) were all significantly higher. The 199 Colilert trays representative of freshwater samples from avian (*n* = 24), dairy (*n* = 48), low impact (*n* = 20), mixed (*n* = 12), sheep and beef (*n* = 44) and urban (*n* = 51) land‐uses, were used for further analysis of 20 *E. coli* isolates from each tray. Molecular typing of the 3980 isolates indicated that B1 was the most common phylotype (1912/3980, 48.04%) followed by almost equal numbers of A (587, 14.75%) and B2 (592, 14.87%) (Figure S1).

**FIGURE 2:**
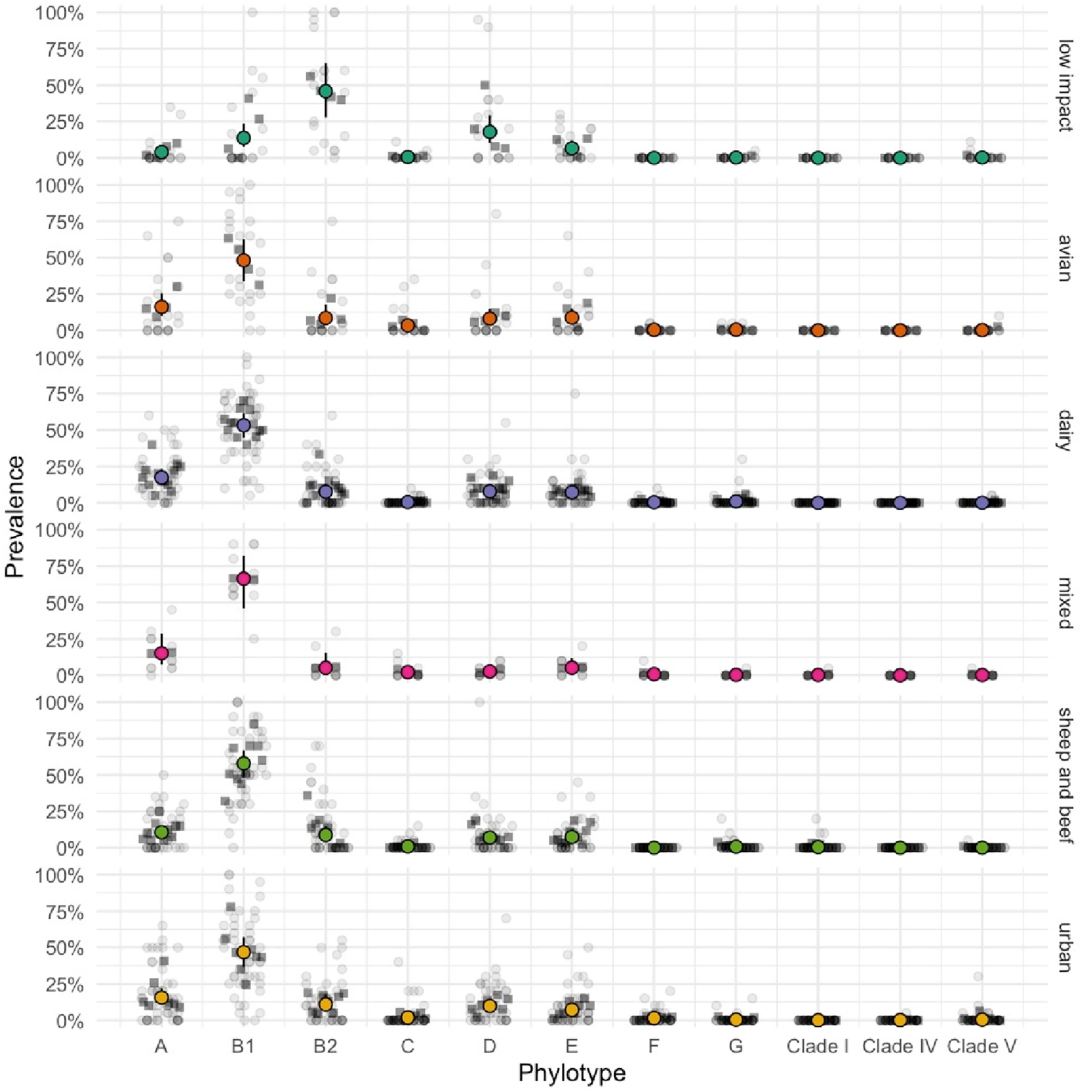
Prevalence of *E. coli* phylotypes and *Escherichia* Cryptic clades from land‐uses. Variation at the individual sample level is represented as light grey circles and individual sites as dark grey squares. Mean abundance of each phylotype for all sites associated with a particular land‐use is represented by the coloured circles with error bars representing 95% confidence intervals. ‘mixed’ relates to land‐use with sheep, beef and dairy farming.

Other *E. coli* phylotypes were less frequently isolated including C (*n* = 67, 1.68%), D (*n* = 409, 10.3%), E (*n* = 319, 8.02%), F (*n* = 24, 0.60%), G (*n* = 34, 0.85%) and Clade I (*n* = 9, 0.23%). Only four isolates were unable to be assigned a phylotype following PCR. Cryptic *Escherichia* were rare, with only one *E. ruysiae*/Clade IV (0.03%) and 22 *E. marmotae*/Clade V (0.55%) identified from 14 sites (1 mixed, 1 avian, 2 dairy, 2 low impact, 8 urban). Phylotype B1 (average 9.6 per sample set of 20 isolates per water sample) isolates were detected at a higher rate than A (average 2.95) and B2 (2.97). Overall, there was more variation of each phylotype (identified from the 20 isolates collected per water sample) related to the number of visits per site compared with variation between the sites with the same land‐use (Figure 2). Overall phylotypes A and B1 were more abundant in the water samples sourced from avian, dairy, mixed, sheep and beef and urban land‐uses whereas the opposite was observed in phylotype B2 which was scarce in the avian, dairy, mixed, sheep and beef and urban land‐uses but much more common in water samples from the low impact sites (Figure 2).

The association between each individual phylotype and land‐use was examined compared to the ‘low impact’ land‐use, where ‘Site’ was included as a random effect. For phylotype A there was a much stronger association with the ‘dairy’ and ‘urban’ land‐use, and B1 across all land‐uses except low impact (Table S2). In contrast, phylotype D, and especially B2 were much less associated with all land‐uses compared to ‘low impact’ (Table S2). Phylotype C was associated with avian, whilst there was no association (*p* > 0.05) between phylotype E and land‐use (Table S2). There was evidence for a difference in phylotype profile by land‐use (F_{pseudo} = 5.01, *p* = 0.0003) and between sites within land‐use (F_{pseudo} = 1.24, *p* = 0.03), with land‐use differences explaining 15.5% of the variation in phylotype profile and site within land‐use explaining 18.2% of variation, leaving 66.2% variation due to visit within site.

When phylotype and *E. coli* log_10_MPN were examined, phylotype A was positively correlated with *E. coli* concentration (log_10_MPN/100 ml) (*p* < 0.0001, OR 1.26, 95% CI 1.17–1.36) for each water sample, but for B2 (*p* = 0.0007, OR 0.86, 95% CI 0.79–0.84) and D (*p* < 0.0001, OR 0.82, 95% CI 0.75–0.90) there was a negative correlation (Table 2). All other phylotypes (B1, E, F and G) were not correlated with *E. coli* concentrations (*p* > 0.05) (Table 2).

**TABLE 2:** Binomial generalized linear mixed effect models were applied to *E. coli* phylotype (20 isolates from each water sample) to examine the association with *E. coli* concentration (log_10_MPN/100 ml).

Phylotype	OR	95% CI	*p*
A	1.26	1.17–1.36	**<0.0001**
B1	1.04	0.98–1.1	0.22
B2	0.86	0.79–0.94	**0.00072**
C	1.13	0.94–1.37	0.19
D	0.82	0.75–0.9	**<0.0001**
E	0.96	0.87–1.05	0.36
F	1.29	0.97–1.72	0.083
G	0.98	0.75–1.28	0.9
Clade I	0.82	0.48–1.41	0.47
Clade IV	1.91	0.61–5.99	0.26
Clade V	0.91	0.63–1.3	0.6

*Note*: *p* < 0.05 in bold. ‘Site’ was included as a random effect.

Abbreviations: OR, odds ratio; 95% CI, 95% confidence intervals.

In addition to culture‐based methods for the detection of *Campylobacter* and *Salmonella*, and microscopy to detect *Cryptosporidium* and *Giardia*, an RT‐PCR‐based approach was undertaken to examine the prevalence of *stx* and *eae* genes in DNA extractions obtained from culture enrichments. Overall *Campylobacter* species were the most common pathogen detected (62.8%, 125 of 199 water samples), followed by the *eae* gene (62.3%, 124 of 199), *Giardia* oocysts (59.8%, 119 of 199) *Cryptosporidium* oocysts (44.2%, 88 of 199), *Salmonella* (24.6%, 49 of 199) and *stx* genes (17.6%, 35 of 199). Although *C. jejuni* (61.3%, 122 of 199) and *C. coli* (18.6%, 37 of 199) were identified separately by PCR, most water samples where *C. coli* was identified (91.4%, 32 of 35) also contained *C. jejuni*, therefore these data were combined to describe the presence of generic *Campylobacter*. At least one of the six pathogen types was detected in 90.0% (179/199) of the water samples; bacteria in 80.9% (*n* = 161) and protozoa in 71.4% (*n* = 142).

The persistence of *E. coli* phylotype B1 has been suggested as an indicator of the age of faecal inputs to a waterbody (Berthe et al., 2013; Ratajczak et al., 2010; Touchon et al., 2020; Walk et al., 2007). The age of faecal contamination was evaluated based on the detection frequency of B1 isolates in each water sample. Recent faecal inputs generally contain a higher diversity of *E. coli* phylotypes and strains (Bergholz et al., 2011; Gordon et al., 2015; Son et al., 2009). To explore the validity of this association between numbers of B1 isolates in a water sample and the age of faecal contamination, samples were grouped to represent low (<10 B1 isolates/20), intermediate (10 to 14 B1 isolates/20), and high (≥15 B1 isolates/20) phylotype B1. Overall, there was no difference of the geometric mean *E. coli* log_10_MPN/100 ml for low (average 344 MPN/100 ml), intermediate (average 557 MPN/100 ml) and high (average 254 MPN/100 ml) B1 isolates (*p* = 0.065). Importantly, increased numbers of individual pathogens (i.e., 1–6) detected were associated with low and intermediate B1 compared to high B1 (*p* < 0.0001). Where B1 were present at <10, 10 to 14, or ≥ 15 isolates, pathogens were present, 88.0% (low B181/92 samples), 98.5% (intermediate B165/66 samples) and 80.5% (high B133/41 samples), respectively (*p* = 0.011). However, although high numbers (≥15 B1 isolates) of persistent phylotype B1 had the lowest number of pathogens on average supporting the hypothesis that persistent high numbers of phylotype B1 could be an indicator of non‐recent, aged contamination events. In addition, a high proportion of these samples with low phylotype B1 had pathogens detected (80.5%) as per the prevalence of ‘Any pathogen’. The presence of *Salmonella*, *eae* gene, *Giardia*, ‘Any bacteria’, and ‘Any protozoa’ were more associated with water samples with low and/or intermediate age B1 levels compared to high (Table S3). Interestingly, ‘Any pathogen’ was associated with intermediate B1 but not low when compared to high numbers (≥15 B1 isolates) of phylotype B1 indicative of non‐recent contamination (Table S3). Neither *Cryptosporidium*, *Campylobacter*, nor *stx* toxin gene presence correlated with the three groupings of B1 persistence (Table S3).

**FIGURE 3:**
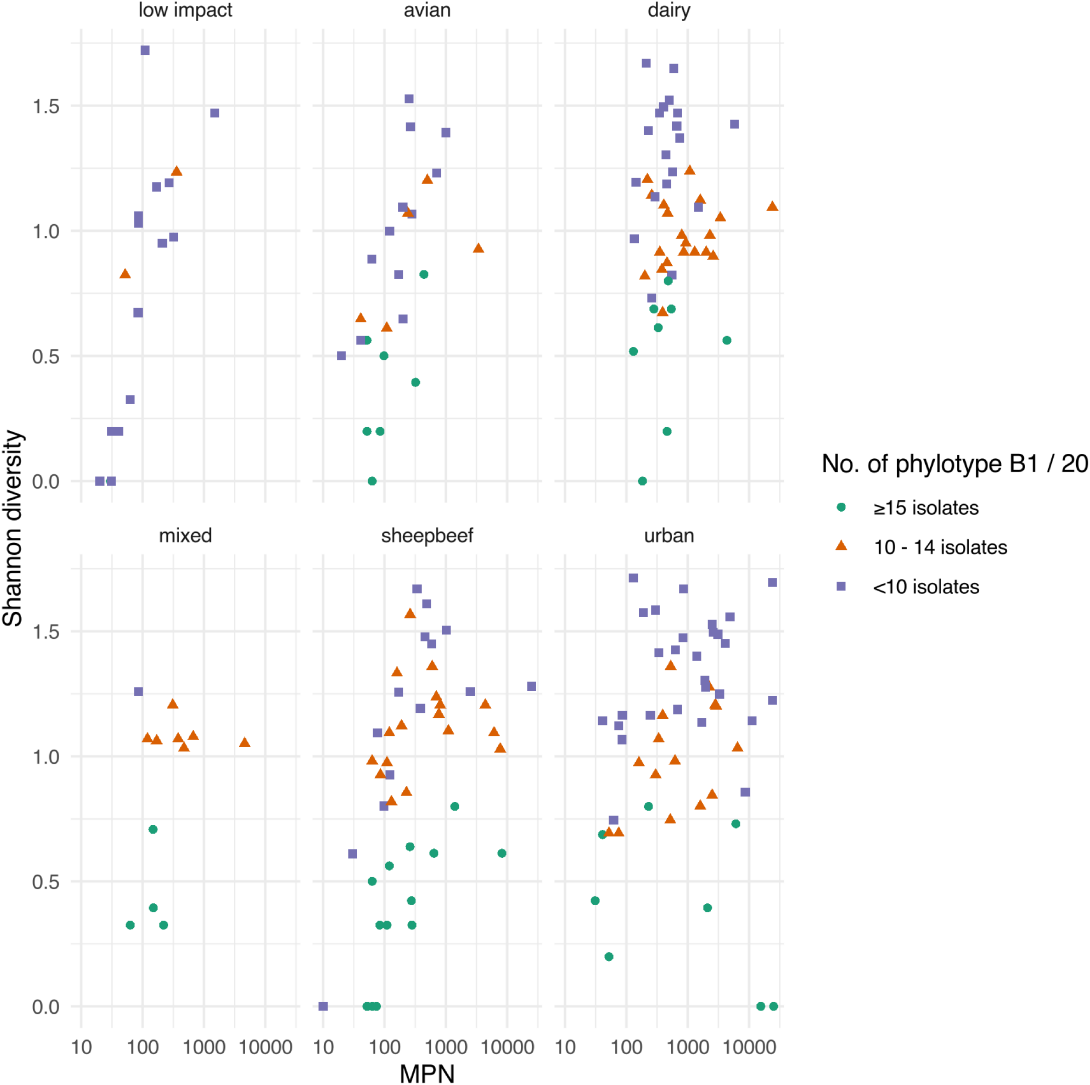
Shannon diversity of phylotypes of 20 *E. coli* isolates obtained from water samples from sites of different land‐use were plotted against *E. coli* concentrations (MPN/100 ml). As a putative indicator of contamination age, data were grouped according to total number of phylotype B1 identified per 20 isolates: Where low represents <10 B1 isolates, 10 to 14 B1 isolates represents intermediate, and high represents ≥15 B1 isolates. ‘mixed’ relates to land‐use with sheep, beef and dairy farming.

The Shannon diversity of phylotypes (per 20 isolates from each water sample) was closely related to *E. coli* log_10_MPN/100 ml (*p* < 0.00001) and compared to high (≥15) phylotype B1, there was increased Shannon diversity in water samples with intermediate (*p* < 0.0001) and low (*p* < 0.0001) B1 (Figure 3). Water samples containing increased phylotype diversity were associated with low (<10) phylotype B1 (Figure 3), but in contrast, water samples from low impact sites where fewer faecal sources would be anticipated were anomalous and characterized by low numbers of phylotype B1 (i.e., <10 B1 isolates/20 isolates) and low phylotype diversity due to increased phylotype B2. Compared to low impact, there was also increased phylotype diversity associated with all other land‐uses (*p* < 0.01). These data suggest that increased *E. coli* concentration (log_10_MPN/ml) increases phylotype diversity, and even after accounting for *E. coli* concentration effects, land‐use and phylotype B1 groupings may also have an effect.

Any link between pathogen detection and phylotype was assessed and for analysis of phylotypes B1, B2, C, D, E, F or G there was no interaction with pathogens (Table S4). However, for phylotype A, there was a positive association with the *eae* gene (*p* = 0.008) and *Salmonella* (*p* = 0.007) (Table S4). Furthermore, *E. coli* concentrations (log_10_MPN/100 ml) were widely associated with bacterial pathogens and diarrhoeagenic *E. coli* genes (Table 3). No interactions were seen with Protozoa, and no negative associations were observed (Table 3).

**TABLE 3:** Binomial generalized linear mixed effect models were applied to pathogen presence/absence data to examine the association with *E. coli* concentration (log_10_MPN/100 ml) where ‘Site’ was included as a random effect.

Association with *E. coli* concentration (log_10_MPN/100 ml)			
Pathogen	OR	95% CI	*p*
Any pathogen	2.95	1.70–5.12	**0.0001**
Any bacteria	2.95	1.86–4.67	**<0.0001**
*Campylobacter*	1.61	1.19–2.19	**0.002**
*eae* gene	3.01	2.06–4.41	**<0.0001**
*Salmonella*	1.44	1.12–1.84	**0.004**
*stx* gene	1.96	1.36–2.82	**0.0003**
Any protozoa	1.16	0.95–1.42	0.15
*Cryptosporidium*	1.09	0.92–1.30	0.32
*Giardia*	1.11	0.93–1.33	0.26

*Note*: ‘Any pathogen’ represents detection of ‘Any bacteria’ and/or ‘Any protozoa’; ‘Any Bacteria’, either *Salmonella*, *Campylobacter*, the *stx* toxin gene or *eae* gene: and ‘Any protozoa’, either *Cryptosporidium* or *Giardia*. *p* < 0.05 in bold.

Abbreviations: OR, odds ratio; 95% CI, 95% confidence intervals.

Detection of pathogens occurred more often from urban and dairy land‐uses compared to low impact sites (Table S5); for avian sites, only the detection of *Salmonella* was more significant from water samples compared to low impact sites (*p* = 0.009). For the *stx* toxin gene, *Giardia* and *Cryptosporidium*, there was no significant association with land‐use compared to low‐impact sites (Table S5).

**FIGURE 4:**
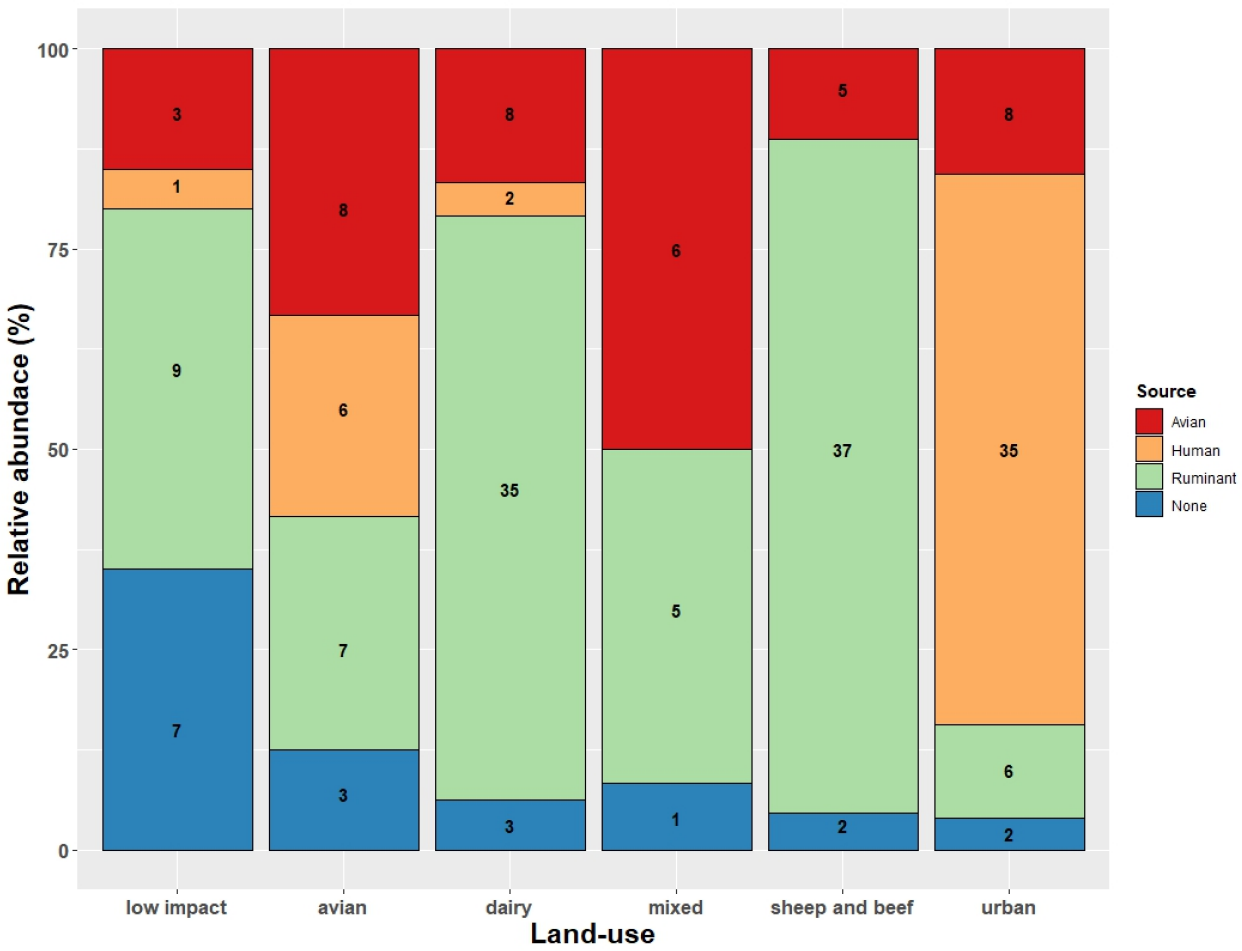
Relative abundance (%) of dominant faecal sources identified within freshwater samples (*n* = 199) collected from different land‐uses. Figures on stacked bars relate to sampling events, that is, number of water samples in each microbial source tracking category.

Three MST markers (Human, CrAssphage, 1.7–6.08 log_10_ Gene Copies [GC]/100 ml; Ruminant, BacR, 1.26–6.46 log_10_ GC/100 ml; Avian, GFD, 1.18–4.15 log_10_ GC/100 ml) were examined in more detail to identify dominant faecal sources associated with land‐use (Figure 4). Most water samples (68.63%, 35 of 51) from urban sites had Human as the dominant faecal source; most from dairy, mixed and sheep and beef sites had Ruminant faecal sources (74.04%, 77 of 104); 33.33% of avian sites (8 of 24) had Avian faecal sources; whereas 35.0%, low impact (7 of 20) had no, or low concentrations, of the faecal sources that were tested, detected.

Although no faecal sources were identified in water samples (18 of 199, 9.04%) with *E. coli* concentrations of between 63 and540 MPN/100 ml, some (10 of 199, 5.02%) had at least one, and up to four different pathogens identified in these water samples.

Avian, Human and Ruminant MST markers (log_10_, GC/100 ml) were examined in more detail to identify associations with land‐use (Table 4), with *E. coli* phylotypes (Table S6) and pathogens (Table 5). Three human MST markers were initially examined, and in subsequent analyses, CrAssphage concentration was used as the human source indicator when affirmed by detection of at least one other human marker detected at the LOQ. In previous study, crAssphage has shown it has a high level of sensitivity and specificity, and persistence in freshwater studies (Ahmed et al., 2021; Ballesté et al., 2019; Gyawali et al., 2021). When compared to low impact areas, faecal source data (log_10_, GC/100 ml) were generally concomitant with land‐use (Table 4); for example, Human MST marker and urban land‐use (*p* < 0.0001), and Ruminant MST marker with dairy land‐use (*p* = 0.004). The Avian MST microbial marker was ubiquitous being found across all land‐uses.

**TABLE 4:** Linear mixed effects models were applied to faecal source data (log_10_, Gene Copies/100 ml water) to examine the association with land‐use, compared to low impact sites.

log_10_ MST marker	Land‐use	Coeff	95% CI	*p*
Ruminant	Low impact	2.27	1.42–3.11	NA
Ruminant	Avian	1.97	1.14–2.80	0.61
Ruminant	Dairy	3.78	3.29–4.27	**0.004**
Ruminant	Mixed	2.90	1.73–4.07	0.38
Ruminant	Sheep and beef	3.80	3.26–4.34	**0.004**
Ruminant	Urban	2.06	1.47–2.64	0.68
Avian	Low impact	1.58	0.96–2.20	NA
Avian	Avian	2.57	1.95–3.18	**0.028**
Avian	Dairy	2.79	2.44–3.15	**0.002**
Avian	Mixed	2.73	1.86–3.60	**0.046**
Avian	Sheep and beef	2.36	1.96–2.76	**0.039**
Avian	Urban	2.90	2.47–3.34	**0.001**
Human	Low impact	1.93	1.22–2.63	NA
Human	Avian	2.26	1.57–2.96	0.49
Human	Dairy	2.25	1.84–2.65	0.43
Human	Mixed	2.27	1.28–3.25	0.57
Human	Sheep beef	2.13	1.68–2.59	0.62
Human	Urban	3.94	3.45–4.43	**<0.0001**

*Note*: ‘Site’ was included as a random effect. Associations *p* < 0.05 are in bold.

Abbreviation: 95% CI, 95% confidence intervals.

**TABLE 5:** Binomial generalized linear mixed effects models were applied to pathogen presence/absence data to examine the association with faecal source data (log_10_, GC/100 ml water) where ‘Site’ was included as a random effect.

Pathogen	log_10_ MST marker	OR	95% CI	*p*
Any pathogen	Ruminant	1.75	0.84–3.67	0.14
Any pathogen	Human	1.78	0.61–5.17	0.29
Any pathogen	Avian	2.53	0.74–8.63	0.14
Any pathogen	GenBac3	1.16	0.58–2.30	0.68
Any bacteria	Ruminant	2.22	1.23–4.01	**0.0082**
Any bacteria	Human	1.84	0.88–3.84	0.1061
Any bacteria	Avian	4.43	1.70–11.50	**0.0022**
Any bacteria	GenBac3	0.61	0.32–1.15	0.1251
*Campylobacter*	Ruminant	1.98	1.22–3.23	0.0058
*Campylobacter*	Human	1.43	0.77–2.64	0.2569
*Campylobacter*	Avian	2.71	1.15–6.41	**0.0228**
*Campylobacter*	GenBac3	0.62	0.33–1.16	0.1371
*eae* gene	Ruminant	2.35	1.54–3.60	**<0.0001**
*eae* gene	Human	1.67	1.01–2.78	**0.046**
*eae* gene	Avian	2.31	1.15–4.64	**0.018**
*eae* gene	GenBac3	0.68	0.40–1.15	0.15
*Salmonella*	Ruminant	1.24	0.84–1.82	0.283
*Salmonella*	Human	1.59	0.94–2.68	0.083
*Salmonella*	Avian	1.39	0.64–3.01	0.411
*Salmonella*	GenBac3	1.05	0.54–2.04	0.887
*stx* gene	Ruminant	2.40	1.56–3.70	**<0.0001**
*stx* gene	Human	1.58	0.95–2.64	0.08
*stx* gene	Avian	0.67	0.31–1.43	0.3
*stx* gene	GenBac3	0.72	0.40–1.30	0.28
Protozoa	Ruminant	1.05	0.76–1.44	0.782
Protozoa	Human	1.26	0.83–1.93	0.281
Protozoa	Avian	1.71	0.94–3.10	0.079
Protozoa	GenBac3	0.91	0.58–1.42	0.682
*Cryptosporidium*	Ruminant	1.29	0.98–1.69	0.071
*Cryptosporidium*	Human	1.22	0.86–1.74	0.268
*Cryptosporidium*	Avian	0.86	0.51–1.46	0.582
*Cryptosporidium*	GenBac3	0.94	0.62–1.42	0.765
Giardia	Ruminant	1.01	0.76–1.34	0.949
Giardia	Human	1.29	0.88–1.88	0.186
Giardia	Avian	1.87	1.08–3.25	**0.026**
Giardia	GenBac3	0.81	0.53–1.24	0.333

*Note*: ‘Any pathogen’ represents detection of ‘Any bacteria’ and/or ‘Any protozoa’; ‘Any Bacteria’, either *Salmonella*, *Campylobacter*, the *stx* toxin gene or *eae* gene: and ‘Any protozoa’, either *Cryptosporidium* or *Giardia*. GenBac3—16S rRNA qPCR positive control marker. Associations *p* < 0.05 are in bold.

Abbreviations: OR, odds ratio; 95% CI, 95% confidence intervals.

*Escherichia coli* phylotype associations with MST markers reflected phylotype and land‐use observations (Table S6). Phylotype A, for example, was more associated with the Avian MST markers (*p* < 0.0001), B1 positively associated with ruminant (*p* = 0.0004), but negatively with Avian (*p* = 0.016) and Human (*p* = 0.005), and B2 negatively with the Ruminant MST marker (*p* = 0.002). Phylotype E was also more commonly associated with the Avian MST marker (*p* = 0.0003), and *E. marmotae* less associated with the Ruminant MST marker (*p* = 0.036). Other phylotypes were not associated with the MST markers (Table S6).

Both bacterial and protozoal pathogens were associated with the three MST markers examined in more detail (Table 5). Despite *Giardia* being associated with the Avian MST marker (*p* = 0.026), there was no association of *Cryptosporidium* with any of the faecal source markers suggesting that oocysts from this protozoan species are equally prevalent and/or ubiquitous with all three MST faecal sources, that is, no discrimination. Notably the zoonotic pathogen *Campylobacter* and the diarrhoeagenic *E. coli*‐associated virulence factors *stx* and *eae* had the highest odds ratio for association with the ruminant faecal source marker (Table 5). The highest odds ratio across all three faecal source markers was for ‘Any bacteria’ with the Avian MST marker (*p* = 0.002, OR 4.43, 95% CI 1.7–11.5). The qPCR 16S rRNA internal control (GenBac3 1.28–7.99 log_10_, GC/100 ml) data was not associated with any of the pathogens examined (Table 5).

The significance of increased rainfall on *E. coli* concentrations (log_10_MPN/100 ml) peaked at 48 h, (24 h, *p* = 0.06; 48 h, *p* = 0.001; 72 h, *p* = 0.09), and with increased flow (*p* = 0.0002). Rainfall was also positively associated with the presence of the *eae* gene (0–24 h: OR 1.18, 95% CI 1.04–1.35, *p* = 0.01), and *stx* genes (0–24 h: OR 1.11, 95% CI 1.04–1.18, *p* = 0.002). Higher flow (preceding 24 h) was significantly associated with increased *E. coli* concentrations (log_10_MPN/100 ml), and there was also a positive association between *Salmonella* (OR 1.04, 95% CI 1.01–1.07, *p* = 0.005) with water flow but no association between ‘Any pathogen’ or the other pathogens detected (*p* > 0.05).

## DISCUSSION

*E. coli* is one of the most‐extensively studied gut bacteria but host/habitat niche specialization (Cookson et al., 2022; Petit et al., 2017; Touchon et al., 2020) of different subtypes has only become apparent through the use of molecular (Clermont et al., 2011; Clermont et al., 2013) and genomic methods (Cookson et al., 2022; Luo et al., 2011; Nowicki et al., 2021) to sub‐type isolates obtained from diverse environments. In parallel, the realization that *E. coli* may also persist in water, soil and sediment has provided new impetus to investigate the maintenance and survival of newly described environmental *E. coli*‐like *Escherichia* spp. (Gilroy et al., 2021; Liu et al., 2015; van der Putten et al., 2021), isolated from terrestrial and aquatic habitats (Koh et al., 2022; Mire et al., 2022; Moinet et al., 2024). The advances in molecular methods to subtype *E. coli* and identify *E. marmotae* and *E. ruysiae* should allow new insights into their overall abundance and association with enteric pathogen species.

This study made use of the routine Colilert (IDEXX) culture enrichments from water samples to provide a comprehensive analysis of *E. coli* phylotypes and recently described *Escherichia* species. Overall, the non‐*E. coli Escherichia* comprised only a minor proportion of the isolates subtyped using PCR (0.6%, 24/3980), with 23 *E. marmotae* and 1 *E. ruysiae* identified from 14 sites (Figure S1). Previous study from New Zealand has indicated that *E. marmotae* and *E. ruysiae* can be isolated from avian faeces, are present in wildlife and pristine bush habitats, and have fewer virulence factors compared to *E. coli* (Cookson et al., 2022; Moinet et al., 2024). Together with data from this study for example, where *E. marmotae* were negatively associated with the Ruminant MST marker (Table S6), these lines of information from recent studies, provide important evidence to water managers that despite being indistinguishable from FIB using standard culture‐based enumeration methods, the recently characterized *Escherichia* species are not associated with waterborne pathogens and represent a lower likelihood of potential health risk to recreational users of water. Furthermore, the low prevalence of these non‐*E. coli Escherichia* species was considerably less than the error of the Quanti‐Tray MPN enumeration method used with the Colilert media (Muirhead & Meenken, 2018). A potential limitation of our experimental approach analysing Colilert enrichments is the impact of numerical dominance of specific *E. coli* clones after incubation and transport (chilled), concomitant with any contrasting competitive exclusion and survival characteristics between different *E. coli* clones in individual Colilert wells. In future studies, more labour intensive, direct recovery of *E. coli* on membrane filters could be employed to assess phylotype variation without sample enrichment. However, we have shown that non‐*E. coli Escherichia* species are unlikely to contribute frequently to FIB exceedances when using Colilert analysis for microbial water quality monitoring.

Here, water samples (*n* = 199) were selected to cover a broad range of *E. coli* concentrations with selection of 50% of samples having *E. coli* concentrations covering the relevant concentrations for recreational water quality guidelines (100–1000 MPN/100 ml). This strategy allowed us to confirm our hypothesis that increased *E. coli* phylotype diversity in each water sample was most likely associated with recent faecal contamination and increased *E. coli* concentrations (Figure 3), however, this was in contrast to a prior study undertaken in an urban area where a negative relationship was observed between *E. coli* concentration and phylotype diversity (Saraceno et al., 2021), likely due to fewer diverse land‐uses and no characterization of the age of faecal contamination events being examined.

What constitutes an aged faecal source and what microbial metrics can be used to help define such a parameter remains unclear. Thus, the extent of this dataset including prevalence of pathogens and breadth of sampling sites provided an opportunity to explore the novel assumption of a candidate microbial metric for faecal age evaluation: high numbers of phylotype B1 in a water sample as suggestive of aged faecal contamination, compared with higher prevalence of other *E. coli* phylotypes (low B1) associated with recent faecal contamination.

Interestingly, water samples dominated by phylotype B1, (potentially) indicative of aged faecal sources, were virtually absent in low impact sites dominated by phylotype B2, likely due to the reduced faecal contamination sources overall (Figure 3). Where low phylotype B1 (potentially) indicative of recent faecal sources were encountered in water from low impact sites, these samples were associated with low *E. coli* concentration. In water sampling sites impacted by reduced faecal sources and with low *E. coli* concentrations, the congruence of low phylotype diversity and the dominance of phylotype B1 likely supports the candidate faecal age metric and reflects the die‐off of other phylotypes less resilient and more unsuited to environmental persistence compared to B1 (Figure 3). Furthermore, water samples impacted by low and/or intermediate B1 levels as a proxy for more recent faecal inputs were associated with an increasing number of bacterial and protozoal pathogen types compared to waters with putative aged sources (high B1). However, this last point needs to be viewed in the context of a high proportion of all samples (≥ 80.5%) containing at least one pathogen.

The generalist nature of the naturalized phylotype B1 and its enhanced environmental resilience (Nowicki et al., 2021; Petit et al., 2017; Touchon et al., 2020) may influence its non‐association with pathogens from more recent faecal contamination events identified in this study (Table S4), but this observation is somewhat confounded by its high prevalence in all land‐uses except low impact. Nevertheless, the low abundance of B1 in low impact areas provides some confidence that these sites were less impacted by faecal sources compared to other land‐uses (Table S2). In the same way, the reduced environmental persistence of phylotype A clearly contrasted with the increased phylotype prevalence data from contrasting land‐uses (e.g., low impact and dairy) (Table S2). Along with B1, phylotype A is considered a generalist often associated with human and animal faecal samples (Johnson et al., 2017; Saraceno et al., 2021) and wastewater (Behruznia & Gordon, 2022; Tavares et al., 2020), and was the only *E. coli* phylotype indicative of recent faecal contamination through a positive association with *E. coli* concentration, bacterial pathogens, but not protozoa.

Other studies have examined the relationship between *E. coli* concentration and pathogens (Hörman et al., 2004; Till et al., 2008; Weller et al., 2020), but none have undertaken a phylotype‐specific approach to examine interactions with common freshwater pathogens such as *Campylobacter*, *Salmonella*, or protozoa (*Cryptosporidium* and *Giardia*). A previous study in Finland failed to identify a significant correlation between *E. coli* and pathogens in water samples taken from rivers and lakes (Hörman et al., 2004) and results may have been impacted by more extreme seasonal temperatures and less variation in the age of faecal sources. In the current study, phylotype B2 was overrepresented in low impact sites (Figure S1 and Table S2), which aligns with our previous observations of its increased abundance in pristine bush environments compared to downstream sites impacted by pastoral farming (Cookson et al., 2022). Intriguingly, phylotype B2 has also been identified as a human‐associated *E. coli* phylotype, commonly isolated from urinary tract infections and associated with the extended‐spectrum beta‐lactamase resistant antibiotic resistance phenotype (Toombs‐Ruane et al., 2023). Therefore, we hypothesise that there are likely to be multiple pathotypes represented by phylotype B2, including those from forested sites (Petit et al., 2017), wildlife (Moinet et al., 2024) and humans with contrasting pathotype‐specific genetic characteristics (Johnson et al., 2017; Smati et al., 2015) which may influence their survival and persistence in the environment. The abundance of B2 in low impact sites less affected by faecal sources in this study contrasts with other studies where B2 were identified as lacking survival‐associated functions and were shown to survive for shorter periods in secondary environments, such as beach sand (Rumball et al., 2020; Rumball et al., 2023). Thus, for environmental persistence studies external to the host, the use of human‐associated phylotype B2 sourced from urban sites or extra‐intestinal *E. coli* pathotypes, may not reflect the survival of B2 sourced from pristine forested sites (Cookson et al., 2022; Petit et al., 2017). Future whole genome sequence analysis and identification of human‐ or environmental‐associated genes from phylotype B2 isolates recovered from contrasting land‐uses in this study will further delineate intra‐phylotype variations and host/niche‐specific characteristics.

In New Zealand, human gastrointestinal disease and notification of infections due to the protozoal pathogens *Cryptosporidium* and *Giardia* is often associated with environmental exposure with recreational water contact being a common risk factor (2020 data: *Cryptosporidium*, 22.5%; *Giardia*, 28.4%; Anon, 2023). Notably, neither protozoal pathogen was associated with *E. coli* concentration (log_10_ MPN/100 ml) (Table 3) or *E. coli* phylotype (Table S4), but ‘Any protozoa’ was positively associated with water samples obtained from dairy land‐use (Table S5), with *Giardia* strongly associated with low B1 (putative recent faecal inputs), and ‘Any protozoa’ with intermediate and low B1 (putative mixed and recent faecal inputs) (Table S3). Both protozoan species have complex life cycles where the infectious oocysts can survive for extensive periods in a freshwater environment prior to subsequent infection of a new host and subsequent waterborne outbreaks of diarrhoeal disease (Devane et al., 2014; Efstratiou et al., 2017). In this study, both *Cryptosporidium* and *Giardia* oocysts were identified from water samples collected from low impact sites, characterized by low *E. coli* concentrations (<50 MPN/100 ml) and suggestive of aged faecal contamination events but which, however, did not match our definition based on B1 abundance (Figure 3). Protozoa, especially *Cryptosporidium*, represent a significant burden of enteric disease in New Zealand with some evidence of seasonal prevalence in waterways (Phiri et al., 2020) corresponding somewhat with human cryptosporidiosis infections (Lake et al., 2008). Despite protozoa detection rates of 71.36% (142/199 water samples), there were fundamental disparities between the association of bacterial pathogens and protozoal oocysts in water column samples and exploratory variables included in this study (e.g., *E. coli* concentration MPN/100 ml, *E. coli* phylotype, faecal age, rainfall, water flow etc.). These observations may be influenced by contrasting kinetics and/or settling velocity deposition through sedimentation (Searcy et al., 2005) for the two microbial types and the contrasting sizes of individual protozoal (oo)cysts (≥5 μm) (Robertson & Lim, 2011; Ryan & Xiao, 2014) and generic *E. coli* cells (1–2 μm). Alternatively, low concentrations in faecal samples (Moriarty et al., 2008) and inability to grow outside hosts, predation of (oo)cysts by ciliated protozoan species (Siqueira‐Castro et al., 2016) or effect of ambient sunlight (Ahmed et al., 2023; King et al., 2008) on oocysts may also influence these disparities.

The use of MST to provide evidence of specific faecal sources provides additional evidence for water managers to apply targeted mitigation strategies to reduce contamination (Devane et al., 2018; Harwood et al., 2014; Tran et al., 2015; Weller et al., 2020). Although dominant Avian faecal sources were widespread in water samples from all land‐uses including low impact (Table 4) and in previous study (Flores et al., 2023), other research using the same avian qPCR (GFD) detected avian faecal sources only rarely (Weller et al., 2020). Comparison of MST markers and *E. coli* phylotype B1 (Table S6) also correlated with the phylotype association with land‐use noted before (Table S2) where phylotype B1 was associated with ruminants (de Castro et al., 2014; Johnson et al., 2017; Massot et al., 2017). Similarly, phylotype A has been found in high proportions in highly polluted urban streams (Saraceno et al., 2021) and was also associated with urban land‐use in this study, but there was no association with the Human MST marker (Table S6).

There was a strong, positive association between bacteria (*Campylobacter*, pathogenic *E. coli stx* and *eae* genes) and the Ruminant MST marker (Table 5). Both *Campylobacter*, such as *C. jejuni* and *C. coli*, and STEC are zoonotic pathogens that commonly inhabit the gut of cattle and sheep as animal reservoirs (Cookson et al., 2006; Irshad et al., 2016). Positive interactions between pathogenic *E. coli* markers and ruminant faecal source markers were noted in previous studies from USA (Weller et al., 2020) and France (Petit et al., 2017), however unlike the study from USA, *eae* was also associated with the Human MST marker (*p* = 0.0(46). Although the *stx* and *eae* genes can be identified together in STEC, these pathogenic *E. coli* markers may also be present separately in STEC/EPEC isolates, including those from freshwater sites (Koczura et al., 2013; Marucci et al., 2011). However, the pathogenicity of water isolates with uncommon *stx*‐subtypes remains to be determined (Cookson et al., 2022) and the *eae* gene has been associated with prolonged EPEC survival in Cladophora (Byappanahalli et al., 2015). Furthermore, although *Campylobacter* was associated with the Ruminant (*p* = 0.006) and Avian (*p* = 0.023) markers, there was no interaction with the Human MST marker (Table 5). This observation may reflect the dominance of pastoral landscapes in the New Zealand environment and the widespread impact of ruminant faecal sources, but that in some extreme cases *Campylobacter* may be associated with human pollution sources in urban areas (Devane et al., 2014). Interestingly *Salmonella* was associated with avian and urban land‐uses but not Human and Avian faecal sources (Table S5 and Table 5). Several pathogenic *Salmonella* serovars have been identified as the zoonotic agent associated with local human disease (Bloomfield et al., 2017; Ford et al., 2019; Fu et al., 2022), and further study is required to establish their prevalence in New Zealand bird species (Moriarty, Karki, et al., 2011a) and links with waterborne outbreaks of disease. Overall, there was some association of pathogens with land‐use that was congruent with MST markers; for example, *Campylobacter* and the *eae* gene were associated with dairy land‐use (Table S5) and the Ruminant MST marker (Table 5).

Freshwater environments and associated *E. coli* concentrations are highly dynamic being influenced by water flow rate and weather events in addition to land‐use (Muirhead & Meenken, 2018). Rainfall events and soil saturation significantly influence the overland flow processes whereby pathogens move across land to water (Muirhead et al., 2004). However, these hydrological events and their impact on the movement of pathogens from soil and faecal material are not uniform, which results in the exceptional variability in multiple factors measured from any particular site in this study. This was illustrated where visit/sampling event was more variable than site in terms of phylotypes identified (Figure 2) and PERMANOVA data where increased phylotype variation was due to visit within site. Combined, these variables interact to significantly influence the *E. coli* concentration, phylotype composition, types and ages of faecal sources, and pathogens detected across different land‐uses. Thus, the composition and metadata associated with every water sample is unique. For example, recent rainfall (48 h preceding sampling) had a significant impact on freshwater *E. coli* concentrations (MPN/100 ml), and the presence of *eae* and *stx* genes as a proxy for pathogenic *E. coli*. Interestingly, the *eae* and *stx* genes were only significantly associated with rainfall in the preceding 24 h of sampling. The contrasting duration of *Campylobacter* (<24 h, *p* = 0.057) and pathogenic *E. coli* after rainfall is likely to reflect both the increased persistence of *E. coli* strains in the environment compared to the more rapid die‐off of *Campylobacter*, such as *C. jejuni* (Moriarty et al., 2012; Moriarty, Mackenzie, et al., 2011b), and the higher concentration of *E. coli* contamination that may occur from multiple animal inputs (Moriarty et al., 2008). Flow measurements were also associated with increased freshwater *E. coli* concentrations (MPN/100 ml), but of the pathogens only *Salmonella*, was associated with increased flow measurements suggesting short‐lived persistence in waterways.

This study took a spatial approach to identify the dominant land‐use associated with freshwater sampling sites, but geo‐spatial mapping often identified multiple land‐use designations for each site (Table S1). Because individual sites are influenced by multiple land‐uses and are likely impacted by multiple faecal sources on any individual water sampling occasion, it is unclear what the relative contribution of land‐use is on pathogen prevalence. However, despite the increased overall *E. coli* MPN/100 ml contamination for dairy (1368 MPN/100 ml) and urban (3347 MPN/100 ml) the prevalence of ‘Any pathogen’ was associated with dairy (*p* = 0.039) but not urban land‐uses (*p* = 0.138) (Table S5). This is supported by another study where *E. coli* and *Campylobacter* were more associated with New Zealand agricultural sites, and protozoa more prevalent in waterways with higher numbers of ruminants in the catchment and lower water quality (Phiri et al., 2020).

Whilst none of the faecal sources monitored were identified from 18 of the water samples, 10 contained at least one and up to four different pathogens. These data clearly indicate that potential recreational health risk remains from, for example, low impact sites, where water samples occur with low *E. coli* concentrations, and where Ruminant, Human or Avian faecal sources are absent. However, a risk assessment would be required to determine if pathogens are present at concentrations likely to affect human health. Analysis with additional MST markers such as cat, dog or possum (*Trichosurus vulpecula*, a prevalent introduced pest) (Moinet et al., 2024) may provide clues on other faecal sources. The influence of sediments on microbial quality in the overlying water column has attracted increasing scrutiny with the proposal that sediments in water bodies can act as reservoirs for both FIB and pathogens (Devane et al., 2014; Devane et al., 2019; Petit et al., 2017). This reservoir effect from non‐recent faecal inputs may be one explanation for the disconnection between MST markers, FIB and pathogen presence in water samples. Persistent faecal microbes may be entrained from sediments into the water column even under base flow conditions. These factors suggest that broad understanding and detailed information regarding catchment dynamics, including temporal and spatial sampling events, are required to characterize the full range of (intermittent) faecal sources likely to affect informed decisions on mitigations.

## CONCLUSION

By taking a molecular approach to delineate *E. coli* and closely related *Escherichia* species (*E. marmotae* and *E. ruysiae*), this study has provided phylotype‐specific associations with dominant land‐use, faecal inputs into a water body and links with bacterial pathogens. Initial observations exploring the *E. coli* population across a wide range of both land‐uses and *E. coli* concentrations, suggests (i) that *E. marmotae*, *E. ruysiae* and *E. whittamii* are rare and not associated with waterborne pathogens, (ii) a clear role for *E. coli* concentrations and phylotype A as an indicator for bacterial freshwater pathogens, (iii) that phylotype B2 is an occupier of low impact sites dominated by exotic/native forest and (iv) that the prevalence of phylotype B1 as a potential metric for faecal age is worthy of additional investigation. Furthermore, compared to studies focussing on highly impacted sites with chronic faecal contamination alone, pathogens were detected from some low impact sites, with low *E. coli* concentrations and an absence of ruminant, avian and human microbial faecal source markers, indicative of potential human health risk. Implementation of new guidelines including these data and further data from the Freshwater Pathogen QMRA study will assist with understanding how many sampling events may be required to understand the dominant faecal sources likely to (intermittently) impact a site. Furthermore, the QMRA modelling will incorporate pathogen concentrations, providing an improved understanding of the potential human health risk associated with recreational water contact.

## AUTHOR CONTRIBUTIONS

**Adrian L. Cookson:** Conceptualization; investigation; funding acquisition; writing – original draft; supervision; data curation; project administration; writing – review and editing; methodology; validation; visualization; formal analysis. **Meg Devane:** Conceptualization; investigation; funding acquisition; methodology; data curation; formal analysis; validation; visualization; writing – original draft; writing – review and editing; supervision; project administration. **Jonathan C. Marshall:** Software; formal analysis; conceptualization; investigation; funding acquisition; writing – review and editing; methodology; validation; visualization; data curation. **Marie Moinet:** Investigation; methodology; formal analysis; project administration; data curation; supervision; writing – review and editing; visualization; validation. **Amanda Gardner:** Investigation; methodology; supervision; writing – review and editing; data curation. **Rose M. Collis:** Investigation; methodology; writing – review and editing; data curation; formal analysis. **Lynn Rogers:** Data curation; formal analysis; methodology; writing – review and editing; investigation. **Patrick J. Biggs:** Writing – review and editing; methodology; investigation; conceptualization; validation; visualization; software. **Anthony B. Pita:** Methodology; writing – review and editing; formal analysis; data curation; investigation. **Angela J. Cornelius:** Methodology; writing – review and editing; formal analysis; data curation; supervision; investigation; validation. **Iain Haysom:** Supervision; resources; writing – review and editing; formal analysis; project administration; data curation; methodology; validation. **David T. S. Hayman:** Writing – review and editing; methodology; supervision; resources; investigation; visualization. **Brent J. Gilpin:** Writing – review and editing; funding acquisition; methodology; validation; project administration; data curation; supervision; resources. **Margaret Leonard:** Funding acquisition; resources; supervision; data curation; project administration; writing – review and editing; methodology; validation.

## CONFLICT OF INTEREST STATEMENT

The authors declare no competing financial interests.

## Supporting information


**DATA S1.** Supporting Information.

**TABLE S1.** Catchment delineation and land‐use identification for freshwater sampling sites. Land cover data (%) for each of the 41 sites was sourced from the Land Cover Database version 5.0, Mainland, New Zealand1. Land‐use Av, avian; D, Dairy; Lm, low impact; Mix, mixed sheep, beef and dairy; Sh, sheep and beef; U, urban.

## Data Availability

The data that support the findings of this study are available on request from the corresponding author.
