# Supplementary material for: Population structure and pathogen interaction of Escherichia coli in freshwater: Implications of land‐use for water quality and public health in Aotearoa New Zealand
Source: Environ Microbiol Rep. 2024 Aug 2;16(4):e13319. doi: 10.1111/1758-2229.13319 (PMC11297283; doi:10.1111/1758-2229.13319)
Supplement: Supplementary file 1 — DATA S1. Supporting Information. [file EMI4-16-e13319-s002.docx]

**Appendix**

**Supplementary Information**

**Material and Methods**

**Figure S1**

**Tables S2 to S6.**

Population structure and pathogen interaction of *Escherichia coli* in freshwater: implications of land-use for water quality and public health in Aotearoa New Zealand.

Adrian L. Cookson^1,2,^*, Meg Devane^3^, Jonathan C. Marshall^4^, Marie Moinet^1^, Amanda Gardner^1^, Rose Collis^1^, Lynn Rogers^1^, Patrick J. Biggs^2,5^, Anthony B. Pita^2^, Angela J. Cornelius^3^, Iain Haysom^3^, David T.S. Hayman^2^, Brent J. Gilpin^3^ and Margaret Leonard^3^.

^1^ AgResearch Limited, Hopkirk Research Institute, Palmerston North, New Zealand.

^2^ mEpiLab, School of Veterinary Sciences, Massey University, Palmerston North, New Zealand

^3^ Institute of Environmental Science and Research, Christchurch, New Zealand

^4^ School of Mathematical and Computational Sciences, Massey University, Palmerston North, New Zealand

^5^ School of Natural Sciences, Massey University, Palmerston North, New Zealand

*** Corresponding author:** Adrian Cookson, AgResearch Limited, Hopkirk Research Institute, Massey University, Tennent Drive, 4442 Palmerston North, New Zealand.

***Materials and Methods.***

***Campylobacter* isolation and detection**

For quantitative analysis of *Campylobacter* by Most Probable Number (MPN) enrichment, water volumes of 1 x 1000 mL, 3 x 10 mL, 3 x 1 mL and 3 x 0.1 mL were analysed (1-3). The 1000 mL and 10 mL aliquots were filtered through 0.22µm (47mm diameter) mixed cellulose ester membranes (Millipore, Merck) and placed into 100 mL and 25 mL of Preston broth (Fort Richard Laboratories Ltd., Auckland, New Zealand), respectively. The 1 mL and 0.1 mL aliquots were directly added to 25 mL Preston broth. Broths were incubated at 41.5 ± 1°C for 24 (±2) h in microaerophilic conditions, and then a loopful plated from each enrichment onto modified charcoal-cefoperazone-deoxycholate agar (mCCDA) plates (Fort Richard). Plates were incubated at 41.5 ± 1°C for 44 (±4) h, and then 2-4 putative *Campylobacter* colonies were transferred to Columbia Blood Agar (CBA) plates (Merck, Darmstadt, Germany) and incubated at 41.5 ± 1°C for 44 h (±4) h. Where putative *Campylobacter* colonies were observed on CBA, conventional PCR using the *Campylobacter* multiplex assay of Wong et al (4) was performed on DNA extracted from the original Preston broth culture with target amplicons of 695 bp for *C. coli*, 246 bp for thermotolerant *Campylobacter* and 99 bp for *C. jejuni*.

***Salmonella* isolation and detection**

For presence/absence analysis of *Salmonella*, 1 L of water was filtered through 0.45 µm (47mm diameter) mixed cellulose ester membrane filters (Millipore, Merck) and placed into 25 mL of buffered peptone water (BPW) broth (Fort Richard Laboratories Ltd). Broths were incubated at 37 ± 1°C for 18 ± 2 h and then 1 mL of BPW culture was transferred into 10 mL of Muller-Kauffmann Tetrathionate Novobiocin Supplement (MKTTn) broth (Fort Richard Laboratories Ltd.), and 0.1 mL of BPW was transferred into 10 mL of Rappaport-Vassiliadis Soya (RVS) peptone broth (Oxoid, Basingstoke, Hampshire, UK), which were then incubated at 37 ± 1°C and 41.5 ± 1°C respectively for 24 ±3 h. A loopful (10 µL) of each broth was plated onto Xylose Lysine Deoxycholate agar (Fort Richard Laboratories Ltd.) and Hektoen Enteric agar (Fort Richard Laboratories Ltd.) and both plates were incubated at 37 ± 1°C for 24 ±3 h. Putative *Salmonella* colonies were inoculated into tryptone broth (Fort Richard Laboratories Ltd.) and urea broth (Fort Richard Laboratories Ltd.), and then plated on MacConkey agar (Fort Richard Laboratories Ltd.) and tryptic soy agar (TSA) (Fort Richard Laboratories Ltd.), and onto both triple sugar iron agar (TSI) (Sigma Aldrich) and Lysine Iron Agar (LIA) slopes (Fort Richard Laboratories Ltd). Isolates that were consistent with *Salmonella* (blackening of slopes) were tested for the presence of *Salmonella* O- and H-antigens by slide agglutination using polyvalent antisera (Remel, Thermo Fisher Scientific, Vilnius, Lithuania). If still indicative of *Salmonella*, biochemical species characterization was undertaken using the Oxoid Microbact GNB 12A and 12B kits in combination (Thermo Fisher Scientific).

***Cryptosporidium* and *Giardia* isolation and detection**

Protozoa were detected according to USEPA Methods (5). Briefly, up to 30 Litres of water was filtered at the sample site through a Filta-Max® filter (IDEXX, Maine, USA) and the filter module was sent overnight at < 10°C to Massey University (Hopkirk Research Institute, Palmerston North) from the Councils. The filter was removed, the foam disks were dismantled and placed into a Stomacher 3500 bag (Seward Ltd, West Sussex, UK) with 500 mL of 1x phosphate buffered saline (PBS). The mixture was homogenised using a Stomacher 3500 (Seward Ltd; West Sussex, UK) for 10 min. The eluent was then transferred into a 500 mL conical centrifuge tube and centrifuged at 1500 x g for 15 min at 10 °C using a Sorvall RT7 Benchtop centrifuge (GMI Inc.; Minnesota, USA). The top 450 mL supernatant was aspirated and discarded. The remaining fluid was vortexed to resuspend the pellet at the bottom of the conical tube and then transferred into a 50 mL falcon tube and centrifuged as before. The supernatant was again aspirated, leaving 10 mL in which the pellet was resuspended. Immunomagnetic separation (IMS) using a Dynabeads GC-Combo kit (Thermo Fisher Scientific) was applied to the sample mixture. These beads are linked to antibodies that recognise a *Cryptosporidium* or *Giardia* surface antigen. The resultant 50 μL fluid was transferred onto a microscope well slide (BioPoint Pty Ltd, Sydney, Australia) and dried at 37°C for 1 h. Then 50 μL of methanol was added to the slide to fix the sample and allowed to dry for 30 min. Immunofluorescent staining was performed using the EasyStain kit (BioPoint Pty Ltd.). A BX 60 fluorescence microscope (Olympus; Tokyo, Japan) was used to scan the slide for *Cryptosporidium* oocysts and *Giardia* cysts. Characteristic features to identify the (oo)cysts and for enumeration was performed as described previously (5).

**Multiplex PCRs for *Escherichia* species and *E. coli* phylotypes**

Each of the boiled lysate preparations (n=3980) were used in individual PCR reactions to determine the *E. coli* phylotype or *Escherichia* cryptic Clades (I to V) using an extended quadruplex PCR phylotype assignment method (6, 7). PCR reactions were carried out in a 20 µL volume containing 10 µL KAPA HiFi HotStart ReadyMix (KAPA Biosystems, Wilmington, United States), 0.3 µM of primer except for TspE4C2 1b and TspE4C2 2b (0.5 µM) and 1 µL of boiled lysate preparation. PCR reactions were performed under the following conditions: 4 min at 94°C, 30 cycles of 20 s at 98°C and 20 s at 61°C, and a final extension for 5 min at 72°C.

Allele specific multiplex PCR for the identification of phylotypes D and E was carried out using primers ArpAgpE.f and ArpAgpE.r (8) and the internal control primers trpBA.f and trpBA.r (9) following the same PCR methods as the quadruplex but with an annealing temperature of 58°C. Allele specific multiplex PCR for phylotypes A and C displaying the +-+- profile in the initial quadruplex PCR phylotype was carried out using custom designed primers dinJF (5’- CCGAATCGATGAAGATCTGAAGAATC) and dinJR (5’- CGCTGTTTTTGATTGATTGAATGGTT) at a final concentration of 0.125 µM; and sorF.F2 (5’- AGCTGGAAAAGTTAGTAAGCATTCAC) and sorF.R (5’- TGTTCACACAGTTCATCAACATTCA) at a final concentration of 0.18 µM, using the following PCR conditions: 5 min at 95°C, 32 cycles of 20 s at 98°C and 20 s at 65°C and a final extension of 72°C for 3 min. Clade I to V PCRs were carried out according to Clermont et al (6) with the following PCR conditions: 4 min at 94°C, 30 cycles of 20 s at 98°C and 20 s at 65.5°C followed by an extension step at 72°C for 5 min. Post-PCR, the amplified DNA fragments were subjected to electrophoresis on a 2% agarose gel. The resultant band patterns were visualised and analysed under UV light after staining with SYBR Safe (ThermoFisher Scientific).

**Real-time PCR (RT-PCR) for detection of diarrhoeagenic *E. coli* genes**

A frozen slurry (400 µL) from each glycerol preparation of pooled Colilert well growth (n=199) was inoculated into 10 mL pre-warmed EC broth (Oxoid) and incubated at 37°C for 3 h with shaking. Broth supernatant was removed by centrifugation (5,000 x g for 10 min at 4°C) and genomic DNA extracted from the cell pellet using the Geneaid Presto Mini gDNA Bacteria kit (dnature, Gisborne, New Zealand).

Real-time multiplex PCR (RT-PCR) assays were performed on an Applied Biosystems® ViiA™7 Real-time PCR System (Life Technologies) targeting the virulence genes *stx*1 and *stx*2 from Shiga toxin-producing *E. coli* (STEC), and intimin adherence (*eae*) gene from enteropathogenic *E. coli* (EPEC) and some STEC, as markers for the presence of pathogenic *E. coli* (10, 11), and the *E. coli* 16S rRNA gene as an internal control. Assays were performed according to the Food Safety and Inspection Service (FSIS) protocol (12) in 25 µL reactions containing 5 µL of purified genomic DNA, 5 µL of PerfeCTa qPCR ToughMix (Quantabio, dnature) with either working stock of 20 or 100 µM primers (dnature) and 5 µM of probe (dnature) as per the FSIS protocol. Positive samples were described as those having quantification cycle thresholds (Cq) of <35.

**Additional metadata**

Rainfall data for water samples in the preceding 24 h, 48 h and 72 h, was recorded by Council staff from each site and visit and was included in analysis. Average flow data (m^3^/s) from the preceding 24 hours prior to sampling were provided by Council staff.

**Microbial source tracking markers**

Up to one litre of freshwater was filtered (in duplicate) through a 0.45 µm mixed cellulose ester membrane filters (Millipore, France). CD1 buffer (800 µL) (PowerSoil Pro kit, Qiagen, Venlo, The Netherlands) was added to the filter and vortexed. Filters were stored at -20°C. Sterile beads (0.5 mm, 11079105, Biospec Products Inc, Oklahoma, US) were added to the filter, and tubes placed in a Minibead beater (Biospec Products), where they were beaten for 3 min at 2,500 rpm, centrifuged at 3,500 g for 5 min and supernatant transferred to a new tube where 200 µL of CD2 buffer (PowerSoil Pro kit, Qiagen) was added, vortexed 5 s, centrifuged at 15,000 g for 1 min. Samples were extracted using the PowerSoil Pro protocol (Qiagen) on the QiaCube extraction robot (Qiagen). If necessary, the volume was adjusted to 650 µL using CD1 buffer (PowerSoil Pro kit, Qiagen) prior to QiaCube extraction. Filters were eluted in 80 µL of elution PowerSoil Pro buffer (PowerSoil Pro kit, Qiagen) and then duplicate filters were combined to a final volume of 160 µL for each water sample (13).

Quantitative PCR (qPCR) analysis was undertaken on a LightCycler 480® (Roche Diagnostics Ltd, California, US) (14). In brief, PCR conditions for the probe-based assays were as follows: Probe based qPCR amplifications were performed in a total volume of 20 µL using 2 µL of DNA template. For GenBac3 and BacR 1 x PerfeCTa qPCR Tough-Mix (Quantabio, Massachusetts, USA), 100 nM of probe, 500 nM of each primer and 0.2 mg/mL of Bovine Serum Albumin (Sigma Aldrich, Burlington, Massachusetts, USA) were used. For the duplex HF183 and crAssphage 1 x PerfeCTa Multiplex qPCR ToughMix (Quantabio, Massachusetts, US), 300 nM of each probe, 600 nM of each primer and 0.2 mg/mL of Bovine Serum Albumin (Sigma Aldrich, Burlington, Massachusetts, USA) were used. SYBR Green based assays used 1 x SYBR Green I Mix (Cat. 04 707 516 001, Roche Diagnostics Ltd); 250 nM of each primer and 0.2 mg/mL of BSA (Sigma Aldrich) with qPCR amplifications performed in a total volume of 25 µL using 2 µL of DNA template.

Thermal cycling conditions for probe-based GenBac3 and BacR assays started with a denaturing cycle of 95°C for 5 min, followed by 45 cycles at 95°C for 10 s and annealing at 60°C for 20 s, and ending with an elongation at 72°C for 10 s. The cycling conditions for the duplex assay started with a denaturing cycle of 95°C for 10 min, followed by 45 cycles at 95°C for 15 s and annealing at 60°C for 60 s. For SYBR green assays, PCR conditions were: a denaturing cycle of 95°C for 5 min, followed by 45 cycles at 95°C for 10 s and annealing at 60°C (BiAdo) or 57°C (GFD) for 10 s, and ending with an elongation at 72°C for 20 s. Melt curves were undertaken for the SYBR green assays using a pre-incubation of 5 minutes at 95°C, followed by 1 minute at 65°C and a ramp rate of 0.11°C to 97°C, with cooling at 40°C for 1 minute.

Standard curves were generated from 10-fold serial dilutions ranging from 10^6^-10^0^ Gene Copies(GC)/100 mL of: gBlock® (Duplex HF183 and crAssphage) (Integrated DNA technologies (IDT), Iowa, US) or Ultramer standards (GFD) (Integrated DNA Technologies, Singapore) or plasmids. Standard curves of plasmid constructs were generated from PCR amplicons of the appropriate target (GenBac3, BacR and BiAdo), cloned into *E. coli* DH5α (Invitrogen, California, USA) using the pGEM-T Easy cloning kit (Promega, Wisconsin, USA). Cq values were translated into GC numbers using single or master standard calibration models (15). A NanoDrop® ND-1000 Spectrophotometer (NanoDrop Technologies, Wilmington, USA), determined the DNA concentration and allowed for calculation of the copy number of target DNA extracts from known length of each gBlock/Ultramer or plasmid construct. The limit of quantification (LOQ) was defined as the lowest standard concentration detected in all three replicates with Cq difference less than 2. The standard concentration detected in two replicates or in three replicates with a Cq difference greater than 2 was considered as a detected but not quantifiable (DNQ) result. Each qPCR run included negative and positive controls, and standard curves of the appropriate target. The amplification efficiency of the PCR assays was considered acceptable at >90% for qPCR targets, and the coefficient of determination (*r*^2^) at ≥0.92 for assays.

***Results.***


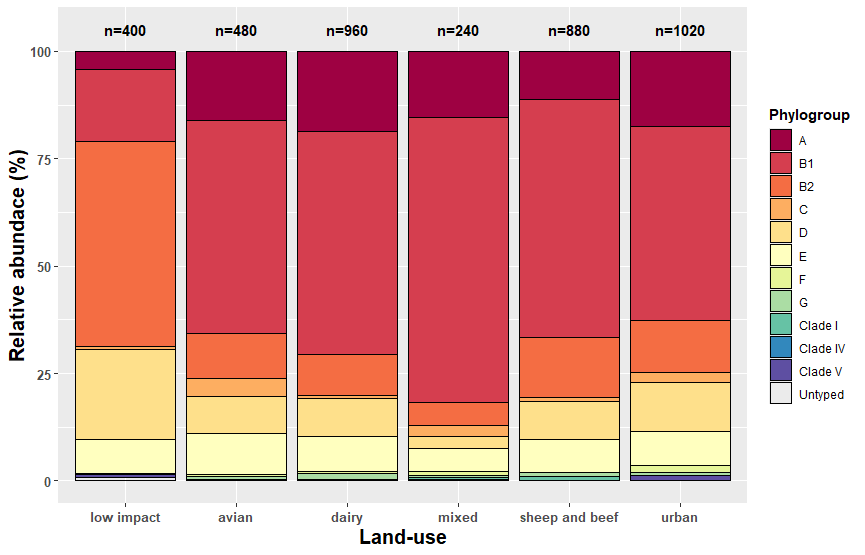


**Figure S1.** Stacked bar plot describing the relative abundance of the different *E. coli* phylotypes and Cryptic clades (Clade I, *E. coli*; Clade IV, *E. ruysiae*; Clade V, *E. marmotae*) identified from water samples obtained from the six different land-uses ('mixed’ relates to sheep, beef and dairy farming).

**Table S2.** Generalised linear mixed effects models were applied to *E. coli* phylotypes to examine the prevalence with land-use compared to respective phylotype abundance from water samples taken from low impact sites where ‘Site’ was included as a random variable. Only phylotypes with > 50 isolates across the 3980 dataset were included. 'mixed’ relates to land-use with sheep, beef and dairy farming. Values are prevalences with 95% confidence intervals in parenthesis. *, P<0.05; **, P<0.01; ***, P<0.001. NA – not applicable.

|  | ***Phylotype (n)*** | |  |  |  |  |
| --- | --- | --- | --- | --- | --- | --- |
| ***Land-use*** | ***A (587)*** | ***B1 (1912)*** | ***B2 (592)*** | ***C (67)*** | ***D (409)*** | ***E (319)*** |
| low impact | 0.04 (0.02 - 0.08) NA | 0.14 (0.08 - 0.23) NA | 0.46 (0.28 - 0.65) NA | 0.01 (0.00 - 0.02) NA | 0.18 (0.10 - 0.29) NA | 0.07 (0.04 - 0.12) NA |
| avian | 0.16 (0.10 - 0.25) ** | 0.48 (0.34 - 0.63) *** | 0.09 (0.04 - 0.18) *** | 0.03 (0.02 - 0.07) * | 0.08 (0.04 - 0.15) | 0.09 (0.05 - 0.15) |
| dairy | 0.17 (0.13 - 0.23) *** | 0.53 (0.45 - 0.62) *** | 0.08 (0.05 - 0.12) *** | 0.01 (0.00 - 0.01) | 0.08 (0.05 - 0.11) * | 0.07 (0.05 - 0.10) |
| mixed | 0.15 (0.07 - 0.29) ** | 0.66 (0.46 - 0.82) *** | 0.05 (0.02 - 0.15) *** | 0.02 (0.01 - 0.07) | 0.03 (0.01 - 0.08) ** | 0.05 (0.02 - 0.12) |
| sheep and beef | 0.11 (0.07 - 0.15) * | 0.58 (0.48 - 0.67) *** | 0.09 (0.05 - 0.15) *** | 0.01 (0.00 - 0.02) | 0.07 (0.05 - 0.11) ** | 0.07 (0.05 - 0.11) |
| urban | 0.16 (0.11 - 0.22) *** | 0.47 (0.37 - 0.57) *** | 0.11 (0.06 - 0.18) *** | 0.02 (0.01 - 0.04) | 0.10 (0.06 - 0.15) | 0.07 (0.05 - 0.11) |

**Table S3.** Binomial generalised linear mixed effects models were applied to pathogen prevalence data to examine the association with low (<10) B1 isolates/20 isolates from each water sample and intermediate (10 to 14) B1 isolates compared to high (≥15) B1 isolates where ‘Site’ was included as a random variable. ‘Any pathogen’ represents detection of ‘Any bacteria’ and/or ‘Any protozoa’; ‘Any Bacteria’, either *Salmonella*, *Campylobacter*, the *stx* toxin gene or *eae* gene; and ‘Any protozoa’, either *Cryptosporidium* or *Giardia*. 'mixed’ relates to land-use with sheep, beef and dairy farming. Associations p<0.05 are in bold. 95% CI, 95% confidence intervals. NA – not applicable.

| ***Pathogen*** | ***B1 isolates*** | ***Prevalence*** | ***95% CI*** | ***P*** | ***Overall P*** |
| --- | --- | --- | --- | --- | --- |
| Any pathogen | high | 0.88 | 0.66 - 0.96 | NA | **0.033** |
| Any pathogen | intermediate | 0.99 | 0.93 - 1.00 | **0.012** | **0.033** |
| Any pathogen | low | 0.95 | 0.81 - 0.99 | 0.143 | **0.033** |
| Any bacteria | high | 0.73 | 0.47 - 0.89 | NA | **0.0035** |
| Any bacteria | intermediate | 0.96 | 0.86 - 0.99 | **0.0015** | **0.0035** |
| Any bacteria | low | 0.93 | 0.79 - 0.98 | **0.0119** | **0.0035** |
| *Campylobacter* | high | 0.54 | 0.29 - 0.76 | NA | 0.13 |
| *Campylobacter* | intermediate | 0.74 | 0.54 - 0.87 | 0.1 | 0.13 |
| *Campylobacter* | low | 0.77 | 0.58 - 0.89 | 0.054 | 0.13 |
| *eae* gene | high | 0.38 | 0.21 - 0.59 | NA | **0.00034** |
| *eae* gene | intermediate | 0.84 | 0.70 - 0.92 | **<0.0001** | **0.00034** |
| *eae* gene | low | 0.66 | 0.50 - 0.79 | **0.016** | **0.00034** |
| *Salmonella* | high | 0.07 | 0.02 - 0.21 | NA | 0.077 |
| *Salmonella* | intermediate | 0.23 | 0.12 - 0.37 | **0.045** | 0.077 |
| *Salmonella* | low | 0.24 | 0.14 - 0.38 | **0.026** | 0.077 |
| *stx* gene | high | 0.12 | 0.05 - 0.26 | NA | 0.21 |
| *stx* gene | intermediate | 0.24 | 0.15 - 0.36 | 0.13 | 0.21 |
| *stx* gene | low | 0.15 | 0.09 - 0.24 | 0.65 | 0.21 |
| Any protozoa | high | 0.56 | 0.40 - 0.71 | NA | **0.048** |
| Any protozoa | intermediate | 0.78 | 0.65 - 0.86 | **0.025** | **0.048** |
| Any protozoa | low | 0.76 | 0.65 - 0.84 | **0.033** | **0.048** |
| *Cryptosporidium* | high | 0.41 | 0.28 - 0.57 | NA | 0.21 |
| *Cryptosporidium* | intermediate | 0.53 | 0.41 - 0.65 | 0.25 | 0.21 |
| *Cryptosporidium* | low | 0.39 | 0.30 - 0.49 | 0.8 | 0.21 |
| *Giardia* | high | 0.44 | 0.30 - 0.59 | NA | 0.055 |
| *Giardia* | intermediate | 0.61 | 0.48 - 0.72 | 0.094 | 0.055 |
| *Giardia* | low | 0.66 | 0.56 - 0.75 | **0.016** | 0.055 |

**Table S4.** Binomial generalised linear mixed effects models were applied to pathogen presence/absence data to examine the association with *E. coli* phylotype/*Escherichia* cryptic clade (20 isolates from each water sample) where ‘Site’ was included as a random variable. ‘Any pathogen’ represents detection of ‘Any bacteria’ and/or ‘Any protozoa’; Any Bacteria’, either *Salmonella*, *Campylobacter*, the *stx* toxin gene or *eae* gene; and ‘Any protozoa’, either *Cryptosporidium* or *Giardia*. Associations p<0.05 are in bold. OR, odds ratio; 95% CI, 95% confidence intervals.

| ***Phylotype*** | ***Pathogen*** | ***OR*** | ***95% CI*** | ***P*** |
| --- | --- | --- | --- | --- |
| A | Any pathogen | 1.23 | 0.97 - 1.56 | 0.089 |
| B1 | Any pathogen | 1 | 0.91 - 1.11 | 0.95 |
| B2 | Any pathogen | 0.96 | 0.86 - 1.07 | 0.41 |
| C | Any pathogen | 313 x10^10^ | 0.00 - Inf | 0.98 |
| D | Any pathogen | 0.93 | 0.82 - 1.06 | 0.3 |
| E | Any pathogen | 1.11 | 0.86 - 1.43 | 0.44 |
| F | Any pathogen | 0.62 | 0.26 - 1.51 | 0.29 |
| G | Any pathogen | 0.99 | 0.43 - 2.32 | 0.99 |
| Clade I | Any pathogen | 3.43x10^9^ | 0.00 - Inf | 1 |
| Clade V | Any pathogen | 0.51 | 0.24 - 1.12 | 0.095 |
| A | Any bacteria | 1.17 | 0.99 - 1.39 | 0.064 |
| B1 | Any bacteria | 0.96 | 0.88 - 1.05 | 0.37 |
| B2 | Any bacteria | 1 | 0.90 - 1.10 | 0.98 |
| C | Any bacteria | 1.05 | 0.64 - 1.73 | 0.85 |
| D | Any bacteria | 0.96 | 0.85 - 1.08 | 0.48 |
| E | Any bacteria | 1.07 | 0.88 - 1.30 | 0.49 |
| F | Any bacteria | 0.9 | 0.38 - 2.12 | 0.81 |
| G | Any bacteria | 1.41 | 0.54 - 3.69 | 0.49 |
| Clade I | Any bacteria | 2.03x10^11^ | 0.00 - Inf | 1 |
| Clade V | Any bacteria | 0.59 | 0.24 - 1.41 | 0.23 |
| A | *Campylobacter* | 1.08 | 0.95 - 1.22 | 0.25 |
| B1 | *Campylobacter* | 0.96 | 0.89 - 1.04 | 0.31 |
| B2 | *Campylobacter* | 1 | 0.90 - 1.10 | 0.95 |
| C | *Campylobacter* | 0.95 | 0.68 - 1.32 | 0.75 |
| D | *Campylobacter* | 1.06 | 0.94 - 1.21 | 0.33 |
| E | *Campylobacter* | 0.99 | 0.84 - 1.16 | 0.9 |
| F | *Campylobacter* | 0.87 | 0.40 - 1.90 | 0.73 |
| G | *Campylobacter* | 0.96 | 0.50 - 1.86 | 0.91 |
| Clade I | *Campylobacter* | Inf | 0.00 - Inf | 1 |
| Clade V | *Campylobacter* | 0.6 | 0.20 - 1.79 | 0.36 |
| A | *eae* gene | 1.17 | 1.04 - 1.32 | **0.0081** |
| B1 | *eae* gene | 0.98 | 0.92 - 1.04 | 0.5 |
| B2 | *eae* gene | 0.96 | 0.89 - 1.05 | 0.38 |
| C | *eae* gene | 1.13 | 0.82 - 1.55 | 0.45 |
| D | *eae* gene | 0.94 | 0.84 - 1.04 | 0.22 |
| E | *eae* gene | 1.06 | 0.91 - 1.22 | 0.45 |
| F | *eae* gene | 1.17 | 0.59 - 2.33 | 0.66 |
| G | *eae* gene | 1.46 | 0.72 - 2.97 | 0.29 |
| Clade I | *eae* gene | Inf | 0.00 - Inf | 1 |
| Clade V | *eae* gene | 0.25 | 0.25 - 0.25 | **<0.0001** |
| A | *Salmonella* | 1.16 | 1.04 - 1.29 | **0.0066** |
| B1 | *Salmonella* | 0.95 | 0.88 - 1.02 | 0.12 |
| B2 | *Salmonella* | 0.93 | 0.84 - 1.04 | 0.19 |
| C | *Salmonella* | 1.28 | 0.95 - 1.71 | 0.11 |
| D | *Salmonella* | 1.04 | 0.94 - 1.16 | 0.45 |
| E | *Salmonella* | 1.03 | 0.89 - 1.19 | 0.72 |
| F | *Salmonella* | 1.17 | 0.59 - 2.29 | 0.66 |
| G | *Salmonella* | 0.37 | 0.09 - 1.55 | 0.17 |
| Clade I | *Salmonella* | 0.73 | 0.18 - 3.01 | 0.66 |
| Clade V | *Salmonella* | 0.86 | 0.40 - 1.87 | 0.71 |
| A | *stx* gene | 1.09 | 0.98 - 1.20 | 0.12 |
| B1 | *stx* gene | 1 | 0.93 - 1.07 | 0.97 |
| B2 | *stx* gene | 0.98 | 0.90 - 1.07 | 0.69 |
| C | *stx* gene | 1.01 | 0.73 - 1.40 | 0.97 |
| D | *stx* gene | 0.93 | 0.81 - 1.08 | 0.34 |
| E | *stx* gene | 1.03 | 0.89 - 1.20 | 0.69 |
| F | *stx* gene | 0.62 | 0.20 - 1.95 | 0.41 |
| G | *stx* gene | 0.82 | 0.40 - 1.68 | 0.58 |
| Clade I | *stx* gene | 0.82 | 0.22 - 3.01 | 0.76 |
| Clade V | *stx* gene | 0.68 | 0.21 - 2.20 | 0.52 |
| A | Any protozoa | 1.03 | 0.93 - 1.15 | 0.52 |
| B1 | Any protozoa | 0.96 | 0.91 - 1.02 | 0.22 |
| B2 | Any protozoa | 1.01 | 0.94 - 1.10 | 0.73 |
| C | Any protozoa | 1.15 | 0.82 - 1.63 | 0.41 |
| D | Any protozoa | 1.01 | 0.91 - 1.11 | 0.9 |
| E | Any protozoa | 1.07 | 0.92 - 1.24 | 0.38 |
| F | Any protozoa | 0.94 | 0.49 - 1.81 | 0.85 |
| G | Any protozoa | 1.19 | 0.67 - 2.10 | 0.56 |
| Clade I | Any protozoa | 0.94 | 0.40 - 2.20 | 0.88 |
| Clade V | Any protozoa | 0.85 | 0.49 - 1.49 | 0.58 |
| A | *Cryptosporidium* | 0.97 | 0.89 - 1.06 | 0.47 |
| B1 | *Cryptosporidium* | 1.03 | 0.98 - 1.08 | 0.26 |
| B2 | *Cryptosporidium* | 0.98 | 0.91 - 1.04 | 0.49 |
| C | *Cryptosporidium* | 1.19 | 0.91 - 1.56 | 0.2 |
| D | *Cryptosporidium* | 0.99 | 0.90 - 1.08 | 0.76 |
| E | *Cryptosporidium* | 0.95 | 0.83 - 1.07 | 0.39 |
| F | *Cryptosporidium* | 1.03 | 0.58 - 1.83 | 0.91 |
| G | *Cryptosporidium* | 1.53 | 0.90 - 2.61 | 0.12 |
| Clade I | *Cryptosporidium* | 1 | 0.45 - 2.22 | 0.99 |
| Clade V | *Cryptosporidium* | 0.71 | 0.35 - 1.45 | 0.35 |
| A | *Giardia* | 1.05 | 0.96 - 1.14 | 0.31 |
| B1 | *Giardia* | 0.95 | 0.90 - 1.00 | 0.054 |
| B2 | *Giardia* | 1.04 | 0.97 - 1.12 | 0.28 |
| C | *Giardia* | 1.17 | 0.87 - 1.57 | 0.31 |
| D | *Giardia* | 0.98 | 0.90 - 1.06 | 0.58 |
| E | *Giardia* | 1.13 | 0.98 - 1.31 | 0.09 |
| F | *Giardia* | 1.17 | 0.63 - 2.16 | 0.63 |
| G | *Giardia* | 0.69 | 0.42 - 1.13 | 0.14 |
| Clade I | *Giardia* | 1.12 | 0.48 - 2.60 | 0.8 |
| Clade V | *Giardia* | 0.92 | 0.54 - 1.56 | 0.75 |

**Table S5.** Binomial generalised linear mixed effects models were applied to pathogen prevalence data to examine the association with land-use compared to respective pathogen prevalence from water samples taken from low impact sites where ‘Site’ was included as a random variable. Any pathogen’ represents detection of ‘Any bacteria’ and/or ‘Any protozoa’; Any Bacteria’, either *Salmonella*, *Campylobacter*, the *stx* toxin gene or *eae* gene; and ‘Any protozoa’, either *Cryptosporidium* or *Giardia*. 'mixed’ relates to land-use with sheep, beef and dairy farming. Associations p<0.05 are in bold. 95% CI, 95% confidence intervals. NA – not applicable.

| ***Pathogen*** | ***Land-use*** | ***Prevalence*** | ***95% CI*** | ***P*** | ***overall P*** |
| --- | --- | --- | --- | --- | --- |
| Any pathogen | low impact | 0.77 | 0.40 - 0.94 | NA | 0.42 |
| Any pathogen | avian | 0.92 | 0.61 - 0.99 | 0.311 | 0.42 |
| Any pathogen | dairy | 0.97 | 0.86 - 1.00 | **0.039** | 0.42 |
| Any pathogen | mixed | 0.94 | 0.51 - 1.00 | 0.32 | 0.42 |
| Any pathogen | sheep and beef | 0.96 | 0.81 - 0.99 | 0.082 | 0.42 |
| Any pathogen | urban | 0.94 | 0.77 - 0.99 | 0.138 | 0.42 |
| Any bacteria | low impact | 0.51 | 0.18 - 0.83 | NA | 0.085 |
| Any bacteria | avian | 0.93 | 0.63 - 0.99 | 0.054 | 0.085 |
| Any bacteria | dairy | 0.98 | 0.87 - 1.00 | **0.0025** | 0.085 |
| Any bacteria | mixed | 0.78 | 0.29 - 0.97 | 0.369 | 0.085 |
| Any bacteria | sheep and beef | 0.87 | 0.66 - 0.96 | 0.067 | 0.085 |
| Any bacteria | urban | 0.86 | 0.63 - 0.96 | 0.087 | 0.085 |
| *Campylobacter* | low impact | 0.24 | 0.05 - 0.66 | NA | 0.088 |
| *Campylobacter* | avian | 0.6 | 0.22 - 0.89 | 0.213 | 0.088 |
| *Campylobacter* | dairy | 0.9 | 0.72 - 0.97 | **0.003** | 0.088 |
| *Campylobacter* | mixed | 0.61 | 0.13 - 0.94 | 0.287 | 0.088 |
| *Campylobacter* | sheep and beef | 0.7 | 0.42 - 0.88 | 0.072 | 0.088 |
| *Campylobacter* | urban | 0.61 | 0.33 - 0.84 | 0.144 | 0.088 |
| *eae* gene | low impact | 0.29 | 0.10 - 0.59 | NA | **0.0064** |
| *eae* gene | avian | 0.5 | 0.25 - 0.76 | 0.292 | **0.0064** |
| *eae* gene | dairy | 0.9 | 0.75 - 0.96 | **0.0003** | **0.0064** |
| *eae* gene | mixed | 0.41 | 0.12 - 0.77 | 0.609 | **0.0064** |
| *eae* gene | sheep and beef | 0.68 | 0.48 - 0.83 | **0.033** | **0.0064** |
| *eae* gene | urban | 0.6 | 0.40 - 0.77 | 0.090 | **0.0064** |
| *Salmonella* | low impact | 0.05 | 0.01 - 0.28 | NA | **0.003** |
| *Salmonella* | avian | 0.5 | 0.29 - 0.71 | **0.009** | **0.003** |
| *Salmonella* | dairy | 0.18 | 0.09 - 0.33 | 0.181 | **0.003** |
| *Salmonella* | mixed | 0.33 | 0.11 - 0.65 | 0.066 | **0.003** |
| *Salmonella* | sheep and beef | 0.09 | 0.03 - 0.22 | 0.572 | **0.003** |
| *Salmonella* | urban | 0.36 | 0.23 - 0.52 | **0.026** | **0.003** |
| *stx* gene | low impact | 0.15 | 0.05 - 0.38 | NA | 0.33 |
| *stx* gene | avian | 0.08 | 0.02 - 0.28 | 0.493 | 0.33 |
| *stx* gene | dairy | 0.19 | 0.10 - 0.32 | 0.712 | 0.33 |
| *stx* gene | mixed | 0.25 | 0.08 - 0.55 | 0.487 | 0.33 |
| *stx* gene | sheep and beef | 0.27 | 0.16 - 0.42 | 0.290 | 0.33 |
| *stx* gene | urban | 0.12 | 0.05 - 0.24 | 0.713 | 0.33 |
| Any protozoa | low impact | 0.55 | 0.34 - 0.75 | NA | 0.14 |
| Any protozoa | avian | 0.63 | 0.42 - 0.79 | 0.615 | 0.14 |
| Any protozoa | dairy | 0.81 | 0.68 - 0.90 | **0.03** | 0.14 |
| Any protozoa | mixed | 0.67 | 0.38 - 0.87 | 0.517 | 0.14 |
| Any protozoa | sheep and beef | 0.66 | 0.51 - 0.78 | 0.405 | 0.14 |
| Any protozoa | urban | 0.8 | 0.67 - 0.89 | **0.034** | 0.14 |
| *Cryptosporidium* | low impact | 0.35 | 0.18 - 0.57 | NA | 0.9 |
| *Cryptosporidium* | avian | 0.38 | 0.21 - 0.58 | 0.86 | 0.9 |
| *Cryptosporidium* | dairy | 0.48 | 0.34 - 0.62 | 0.33 | 0.9 |
| *Cryptosporidium* | mixed | 0.42 | 0.18 - 0.69 | 0.71 | 0.9 |
| *Cryptosporidium* | sheep and beef | 0.48 | 0.34 - 0.62 | 0.34 | 0.9 |
| *Cryptosporidium* | urban | 0.45 | 0.32 - 0.59 | 0.44 | 0.9 |
| *Giardia* | low impact | 0.5 | 0.29 - 0.71 | NA | 0.59 |
| *Giardia* | avian | 0.54 | 0.35 - 0.73 | 0.78 | 0.59 |
| *Giardia* | dairy | 0.58 | 0.44 - 0.71 | 0.53 | 0.59 |
| *Giardia* | mixed | 0.58 | 0.31 - 0.82 | 0.65 | 0.59 |
| *Giardia* | sheep and beef | 0.57 | 0.42 - 0.70 | 0.61 | 0.59 |
| *Giardia* | urban | 0.71 | 0.57 - 0.81 | 0.11 | 0.59 |

**Table S6.** Linear mixed effects models were applied to *E. coli* phylogroups/*Escherichia* cryptic clades to examine the association with faecal source data (log_10_, Gene Copies /100 mL water) where ‘Site’ was included as a random variable. GenBac3 - 16S rRNA qPCR positive control marker. Associations p<0.05 are in bold. OR, odds ratio; 95% CI, 95% confidence intervals.

| ***Phylotype*** | ***log_10_ MST marker*** | ***OR*** | ***95% CI*** | ***P*** |
| --- | --- | --- | --- | --- |
| A | Ruminant | 0.97 | 0.88 - 1.08 | 0.59 |
| A | Human | 1.14 | 0.99 - 1.33 | 0.075 |
| A | Avian | 1.68 | 1.35 - 2.08 | **<0.0001** |
| B1 | Ruminant | 1.18 | 1.08 - 1.30 | **0.0004** |
| B1 | Human | 0.82 | 0.71 - 0.94 | **0.005** |
| B1 | Avian | 0.81 | 0.68 - 0.96 | **0.016** |
| B2 | Ruminant | 0.8 | 0.70 - 0.92 | **0.002** |
| B2 | Human | 1.19 | 0.98 - 1.45 | 0.077 |
| B2 | Avian | 0.84 | 0.66 - 1.07 | 0.15 |
| C | Ruminant | 0.95 | 0.75 - 1.21 | 0.68 |
| C | Human | 1.25 | 0.88 - 1.79 | 0.21 |
| C | Avian | 1.42 | 0.86 - 2.35 | 0.17 |
| D | Ruminant | 1.03 | 0.90 - 1.18 | 0.65 |
| D | Human | 1.02 | 0.85 - 1.23 | 0.81 |
| D | Avian | 0.97 | 0.76 - 1.23 | 0.79 |
| E | Ruminant | 0.94 | 0.83 - 1.06 | 0.29 |
| E | Human | 0.84 | 0.70 - 1.01 | 0.058 |
| E | Avian | 1.55 | 1.22 - 1.97 | **0.0003** |
| F | Ruminant | 0.67 | 0.41 - 1.09 | 0.11 |
| F | Human | 1.59 | 0.89 - 2.83 | 0.12 |
| F | Avian | 0.78 | 0.32 - 1.90 | 0.58 |
| G | Ruminant | 0.91 | 0.61 - 1.34 | 0.62 |
| G | Human | 0.76 | 0.44 - 1.30 | 0.31 |
| G | Avian | 0.62 | 0.31 - 1.21 | 0.16 |
| Clade I | Ruminant | 2.52 | 2.51 - 2.54 | **<0.0001** |
| Clade I | Human | 0.33 | 0.33 - 0.33 | **<0.0001** |
| Clade I | Avian | 1.86 | 1.85 - 1.87 | **<0.0001** |
| Clade V | Ruminant | 0.54 | 0.30 - 0.96 | **0.036** |
| Clade V | Human | 1.1 | 0.59 - 2.05 | 0.759 |
| Clade V | Avian | 1.93 | 0.77 - 4.84 | 0.16 |

***References***

1. APHA. 2017. Detection of pathogenic bacteria, 9260 G *Campylobacter*. Standard methods for the examination of water and wastewater. American Public Health Association, Washington, DC.,

2. Medeiros D, Hoffman L. 2002. Isolation of Campylobacter from food, MFLP-46, Compendium of analytical methods. Health Canada, Ontario.

3. ISO. 2017. ISO 10272-1:2017 microbiology of the food chain — horizontal method for detection and enumeration of *Campylobacter* spp. — part 1: Detection method. International Organization for Standardization.

4. Wong T, Devane M, Hudson JA, Scholes P, Savill M, Klena J. 2004. Validation of a PCR method for *Campylobacter* detection on poultry packs. British Food Journal 106:642-650.

5. USEPA. 2005. Method 1623: Cryptosporidium and Giardia in Water by Filtration/IMS/FA. EPA 815-R-05-002. Agency. WDUSEP,

6. Clermont O, Gordon D, Brisse S, Walk S, Denamur E. 2011. Characterization of the cryptic *Escherichia* lineages: rapid identification and prevalence. Environmental Microbiology 13:2468-2477.

7. Clermont O, Christenson J, Denamur E, Gordon D. 2013. The Clermont *Escherichia coli* phylo-typing method revisited: improvement of specificity and detection of new phylo-groups. Environmental Microbiology Reports 5:58-65.

8. Lescat M, Clermont O, Woerther PL, Glodt J, Dion S, Skurnik D, Djossou F, Dupont C, Perroz G, Picard B, Catzeflis F, Andremont A, Denamur E. 2012. Commensal *Escherichia coli* strains in Guiana reveal a high genetic diversity with host-dependant population structure. Environmental Microbiology Reports 5:49-57.

9. Clermont O, Lescat M, O'Brien CL, Gordon DM, Tenaillon O, Denamur E. 2008. Evidence for a human-specific *Escherichia* coli clone. Environmental Microbiology 10:1000-1006.

10. Nataro JP, Kaper JB. 1998. Diarrheagenic *Escherichia coli*. Clinical Microbiology Reviews 11:142-201.

11. Clarke SC. 2001. Diarrhoeagenic *Escherichia coli*—an emerging problem? Diagnostic Microbiology and Infectious Disease 41:93-98.

12. FSIS. 2019. PCR Platform Instructions for the real-time PCR detection of Shiga toxin gene and H7 gene in *E. coli* O157:H7. United States Department of Agriculture FSIS, Office of Public health Science, Athens, GA. <https://www.fsis.usda.gov/sites/default/files/media_file/2021-03/mlg-5-appendix-5.pdf>.

13. Gilpin B, Hewitt J, Scholes P, Coxon S, Hayman DTS, Pita AB, Knox M, Garcia-Ramirez JC. 2018. Freshwater Microbiological Sciences Review (FMSR): Stage One. Work stream B: Microbial methods. Report CSC18018. One). PfMftEapotFMSRS,

14. Devane M, Robson B, Nourozi F, Wood D, Gilpin BJ. 2013. Distinguishing human and possum faeces using PCR markers. Journal of Water and Health 11:397-409.

15. Sivaganesan M, Haugland RA, Chern EC, Shanks OC. 2010. Improved strategies and optimization of calibration models for real-time PCR absolute quantification. Water Research 44:4726-4735.
